# Photoplethysmogram Analysis and Applications: An Integrative Review

**DOI:** 10.3389/fphys.2021.808451

**Published:** 2022-03-01

**Authors:** Junyung Park, Hyeon Seok Seok, Sang-Su Kim, Hangsik Shin

**Affiliations:** ^1^Department of Biomedical Engineering, Chonnam National University, Yeosu, South Korea; ^2^Department of Convergence Medicine, University of Ulsan College of Medicine, Asan Medical Center, Seoul, South Korea

**Keywords:** bio-signal processing, motion artifacts, photoplethysmography, physiological signal, signal quality assessment, noise reduction, physiological measurement

## Abstract

Beyond its use in a clinical environment, photoplethysmogram (PPG) is increasingly used for measuring the physiological state of an individual in daily life. This review aims to examine existing research on photoplethysmogram concerning its generation mechanisms, measurement principles, clinical applications, noise definition, pre-processing techniques, feature detection techniques, and post-processing techniques for photoplethysmogram processing, especially from an engineering point of view. We performed an extensive search with the PubMed, Google Scholar, Institute of Electrical and Electronics Engineers (IEEE), ScienceDirect, and Web of Science databases. Exclusion conditions did not include the year of publication, but articles not published in English were excluded. Based on 118 articles, we identified four main topics of enabling PPG: (A) PPG waveform, (B) PPG features and clinical applications including basic features based on the original PPG waveform, combined features of PPG, and derivative features of PPG, (C) PPG noise including motion artifact baseline wandering and hypoperfusion, and (D) PPG signal processing including PPG preprocessing, PPG peak detection, and signal quality index. The application field of photoplethysmogram has been extending from the clinical to the mobile environment. Although there is no standardized pre-processing pipeline for PPG signal processing, as PPG data are acquired and accumulated in various ways, the recently proposed machine learning-based method is expected to offer a promising solution.

## Introduction

**FIGURE 1:**
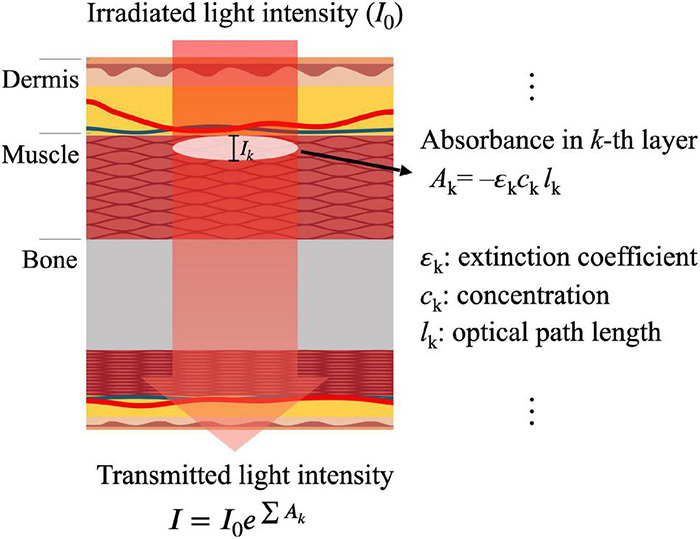
Light intensity change represented with the Beer–Lambert law in photoplethysmogram measurement, where *A*_k_, ε_k_, *c*_k_, and *l*_k_ are the *k*-th layer absorbance, extinction coefficient, concentration, and optical path length, respectively.

Photoplethysmography (PPG) is a non-invasive method for measuring blood volume changes in a microvascular bed of the skin based on optical properties, such as absorption, scattering, and transmission properties of human body composition under a specific light wavelength (Challoner, 1979). PPG is a compound word that consists of “photo,” meaning light; “plethysmo,” meaning volume; and “graphy,” meaning recording (Alnaeb et al., 2007). In 1937, Hertzman found that the amount of light detected by back scattering after irradiating light to the skin was significantly changed according to cardiac activity. He suggested that PPG was a technique for measuring blood volume changes in a specific area irradiated with light (Hertzman, 1937, 1938). PPG records the amount of light transmitted or reflected by the change in concentration of substances in the blood and the optical path according to pulsation, which can be explained by the Beer–Lambert law that defines the attenuation of light intensity by the extinction coefficient, concentration, and optical path length of a medium when light passes through it (Beer, 1851). The Beer–Lambert law, as shown in*I* = *I*_0_*e*^−ε*lc*^, defines that the transmitted light intensity (*I*) through a medium will decrease exponentially in irradiated light intensity (*I*_0_) in relation to the absorption coefficient (ε), optical path length (*l*), and concentration of the medium (*c*). The exponent part of the Beer–Lambert law is defined as absorbance (A), which can be expressed as *A* = −ε*lc*. The Beer–Lambert law is used in various PPG applications that include calculating oxygen saturation (Nitzan et al., 2014) and developing multi-layer light–skin interaction models (Liu et al., 2016a). A recent study, based on modified Beer–Lambert law, measured PPG depending on skin depth by applying different extinction coefficients according to characteristics of the microvascular bed of the skin (Baker et al., 2014; Liu et al., 2016a). Figure 1 shows skin structure, optical path, and light intensity change represented by the Beer–Lambert law in photoplethysmogram measurement. Light irradiated into the skin will pass through skin structures, such as tissues, veins, and arteries; then, finally it is detected by a photodetector. The amount of light absorbed or scattered during this process may vary depending on the composition of the skin structure. In Figure 1, the total absorbance throughout skin layers is equal to the total sum of the absorbances of the *k* layers (*A*_k_ = −ε_k_*c*_k_*l*_k_), where ε, *c*, and *l* are the extinction coefficient, concentration, and optical path length, respectively, and the amount of light that is finally transmitted can be expressed as *I* = *I*_0_^*e*∑*A*_k_^. In this case, the total absorbance depends on the skin structure.

Unlike transmissive-mode PPG that has a straight optical pathway, reflective-mode PPG requires a more complex physical model, because the optical path between the emitter and the photodetector is curved and has a nonlinear pathway. Rubynok and Kyriacou assumed that the optical path between the emitter and the photodetector has multiple “canoe” shapes and modeled the absorbance of the Beer–Lambert law *A*_*piλ*_ as $A_{pi\lambda }=-\log\left(I_{Dpi\lambda }/I_{Epi\lambda }\right)=\mu_{\lambda }l_{pi\lambda }=\sum_{j=0}^{m_{\lambda }}\mu_{sj\lambda }l_{pisj\lambda }$through the banana-shaped mean light pathway representing each “canoe” shape (Rybynok and Kyriacou, 2010), where, *I*_*Dpiλ*_ and *I*_*Epiλ*_ are the radiation and detection light intensity, respectively, in the banana-shaped light pathway, μ_λ_ is the absorption coefficient for the whole optical pathway in the scattering sample, *l*_*piλ*_ is the mean optical pathway corresponding to the pi fraction of the transmitted light power in the vascular tissue, *m* is the matter segments along the mean light pathway with different absorption coefficients, μ_*sjλ*_ is the total absorption coefficient tilde within the scattering matter segment *sj*, and *l*_*pisjλ*_ is a part of the mean light pathway within the scattering matter segment *sj*. The total absorption coefficient μ_*sjλ*_ can be further extended by absorptivities and concentrations of the absorbing components present in the light pathway segment *sj*:$\mu_{sj\lambda }=\sum_{k=0}^{n_{sj}}\varepsilon_{k\lambda }c_{ksj}$, where *n*_*sj*_ is the number of light-absorbing components in the *j*, ε_*kλ*_is the Beer–Lambert law absorptivity of the absorbing component *k* at wavelength λ, and *c*_*ksj*_ is the concentration of the light absorbing component *k* in the light pathway segment *j*. In addition, the extinction coefficient of the reflective mode can be modeled as Δ*A* = *log*⁡(Δ*I*_0_/Δ*I*) = εΔ*cl*⋅*DPF* through the differential path length factor (DPF) based on the Modified Beer–Lambert Law (MBLL), where $DPF\left(\lambda \right)\approx \frac{1}{2}{\left(3\mu_{s}^{\prime }\left(\lambda \right)/\mu_{a}\left(\lambda \right)\right)}^{1/2}$ and μ_*a*_(λ) and $\mu_{s}^{\prime }\left(\lambda \right)$ are the absorption coefficient and reduced scattering coefficient, respectively (Pintavirooj et al., 2021).

The volume of blood volume in the measurement site, arterial diameter, hemoglobin concentration, and hemoglobin direction according to the cardiac cycle are also major factors that affect the detected light intensity (De Trafford and Lafferty, 1984; Kamal et al., 1989; Lindberg and Oberg, 1993). For example, during the diastolic phase, blood volume, arterial diameter, and hemoglobin concentration in the measurement site are minimized. Thus, absorbance is minimized, while the amount of light detected by the photodetector is maximized. Conversely, in the systolic phase, the light intensity detected by the photodetector becomes minimum (Ding and Zhang, 2015; Ding et al., 2017).

Photoplethysmography (PPG) can be measured using light sources of various wavelengths. In general, when the wavelength of light increases, the depth of penetration also increases (Spigulis et al., 2007a,b; Ruggiero et al., 2016). For example, it is known that wavelengths of 470, 570, and 660 nm or more can reach the epidermis with capillaries, dermis with arterioles, and arteries of subcutaneous tissues, respectively (Liu et al., 2015, 2016a,2016b,^2018^). Major blood vessels and arteries with strong pulsation are mainly located in the skin dermis or subcutaneous tissue. Thus, light with a red wavelength of 640–660 nm and infrared wavelength of 880–940 nm is mainly used for PPG measurement (Jones, 1987). PPG is mainly obtained at the extremities of the human body, such as fingers, toes, and earlobes that are advantageous for measuring changes in blood volume, because the vascular bed is shallow and widely spread (Stern, 1974; Allen and Murray, 2002; Millasseau et al., 2006). PPG can also be obtained from the forehead, esophagus, and nose (Barnes et al., 1977; Kyriacou et al., 2002; Choi et al., 2018).

**FIGURE 2:**
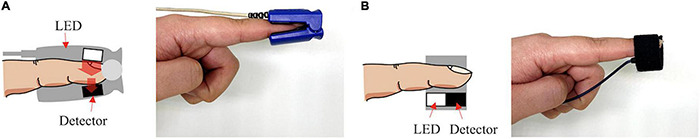
Configuration for photoplethysmography measurement: **(A)** transmissive type and **(B)** reflective type.

A PPG device is composed of a light-emitting diode (LED) that emits light, and a photodetector that detects the emitted light. The device can be divided into transmissive type and reflective type according to the position of the LED and photodetector. Figure 2 shows configurations for a photoplethysmogram measurement device. For the transmissive type, the photodetector is located on the opposite side of the LED, with skin tissues in between. For the reflective type, the photodetector is located next to the LED. Since the transmissive type measures attenuated light intensity after the light passes through skin tissues, it is mainly used for measuring PPG in the distal part of the body, where skin tissues, such as those of fingers, toes, and earlobes, are thin. The transmission-type PPG sensor shows more stable PPG measurement performance than the reflective type (Li et al., 2018). On the other hand, since the reflective type measures scattered light intensity after light irradiates the skin tissue, the measured light intensity is relatively smaller than that of the transmissive type, and the quality of the signal may be degraded. However, it has the advantage of being able to measure PPG not only in the distal part of the body but also in parts of the body, such as the forehead, wrist, carotid artery, and esophagus, where light transmission is difficult (Venema et al., 2012; Wannenburg and Malekian, 2015). The PPG measurement system has the basic hardware structure of an LED to irradiate light, and a photo detector to measure the amount of transmitted light; in addition, it includes an emitter driver to drive the LED, a filter to remove noise and enhance the quality of the obtained signal, an analog-to-digital converter, and a microprocessor. Due to its low cost and a simple hardware structure characteristic, PPG has been used in various applications.

In a clinical environment, PPG is typically used for measuring blood oxygen saturation (pulse oximetry), peripheral vascular tone, and changes in peripheral blood flow according to the respiratory cycle. Blood oxygen saturation is calculated as the ratio of the concentration of oxyhemoglobin to total hemoglobin in the blood. Traditionally, both infrared wavelength (∼880 nm) and red wavelength (∼660 nm) are used for measuring oxygen saturation, because deoxyhemoglobin absorbs more red wavelength, while oxygenated hemoglobin absorbs more infrared wavelength (Zijlstra et al., 1991; Webster, 1997; Sinex, 1999).

Perfusion index measured with PPG is defined as the ratio of pulsatile component to non-pulsatile component of PPG. It indicates the contraction of peripheral vascular smooth muscle. It is used for peripheral vascular tone evaluation related to hypertension and coronary artery diseases (Shelley et al., 1997; Hummler et al., 2006; Landsverk et al., 2008; Mowafi et al., 2008, 2009). The Pleth variability index indicates the fluctuation of perfusion index, which is known to have an inverse relationship with blood flow in blood vessels (Cannesson et al., 2008b; Zimmermann et al., 2010). Changes in blood flow in peripheral blood vessels according to the respiratory cycle can be measured to monitor patients with respiratory distress or heart failure; this technique is also used to evaluate the intrathoracic pressure–cardiac function correlation (Cannesson et al., 2005; Monnet et al., 2005). PPG is also used in arterial blood pressure estimation, heart function evaluation, and pain assessment studies. Using PPG, arterial blood pressure can be estimated by hemodynamic modeling (Chen et al., 2000; Poon and Zhang, 2005). It can also be estimated using a linear or nonlinear regression model based on pulse transit time (PTT) derived by PPG (Fung et al., 2004; Muehlsteff et al., 2006; Baek et al., 2009; Wong et al., 2009; Mase et al., 2011; Ma, 2014; Mousavi et al., 2019). In pain assessment research using PPG, the surgical Pleth index (SPI; GE Healthcare, Chicago, IL, United States) has been calculated through the amplitude and heart beat interval of PPG for intraoperative pain evaluation (Ahonen et al., 2007; Struys et al., 2007; Kallio et al., 2008). Another study has shown that the amplitude variation, area, triangulated area, width, ascending slope, and descending slope of PPG are significantly correlated with pain (Yang et al., 2018; Seok et al., 2019). PPG measured with a mobile device can be used to evaluate the exercise state, sleep state, and stress index of a user through various approaches based on pulse rate and respiratory rate analysis or waveform analysis (Choi et al., 2011; Lin et al., 2011; Madhav et al., 2011; Karlen et al., 2013; Parak and Korhonen, 2014; Temko, 2017; Zangróniz et al., 2018; Saganowski et al., 2020). Compared with other hemodynamic analysis devices, PPG is an inexpensive and noninvasive technique with higher mobility. It is also an easy technique for attaching electrodes and measuring signals. Thus, its use in the clinical and mobile fields is increasing. However, PPG is easily affected by various external factors, such as the body temperature of the measurement site (Senay et al., 1963; Bohusch et al., 1994), intensity of ambient light in the experimental space (Kim et al., 2015), and individual differences, such as skin type (Adler et al., 1998; Spigulis et al., 2007a; Fallow et al., 2013); therefore, additional research on advanced signal processing techniques is needed to obtain a robust PPG waveform.

The purpose of this study was to examine PPG from an engineering viewpoint through the previous research and literature, and review the current status and vision of PPG, including its measurement principle and mechanism, waveform characteristics, representative noise, pre-processing technology, feature extraction technology, and post-processing technology. Reviewing the results of the research performed to date on the above contents is expected to contribute to the application of PPG, which, with the recent growth of mobile healthcare for daily health care or clinical environment, is increasingly being utilized.

## Methods

### Search Strategy

A review of the literature was conducted using the following five databases: PubMed, Institute of Electrical and Electronics Engineers (IEEE), Google Scholar, ScienceDirect, and Web of Science. Search terms *photoplethysmogram*, *review*, *motion artifacts (MA)*, *preprocessing, signal processing*, *noise reduction*, *derivative*, *feature*, *feature detection*, *peak*, *peak detection*, *noise*, *waveform*, *signal quality*, and *perfusion* were combined.

### Inclusion Criteria

To be eligible for inclusion in this review, the primary requirement was that an article needed to focus on signal characteristics, waveform analysis, noise reduction, peak detection, waveform reconstruction, or quality assessment of PPG. If possible, the literature review was focused on recently published articles or articles with a high number of citations, but reports were not excluded because of their year of publication. However, review articles and original articles not published in English were excluded.

### Review Process

The searched articles were reviewed, and detailed subcategories were organized according to the characteristics and processing procedures of PPG. In this process, the authors selected appropriate articles focusing on subcategories, and detailed technological items were listed through in-depth review. In discussion, all the authors presented the details and trends of subcategories and drew conclusions based on common trends.

## Results

### Photoplethysmogram Waveform

**FIGURE 3:**
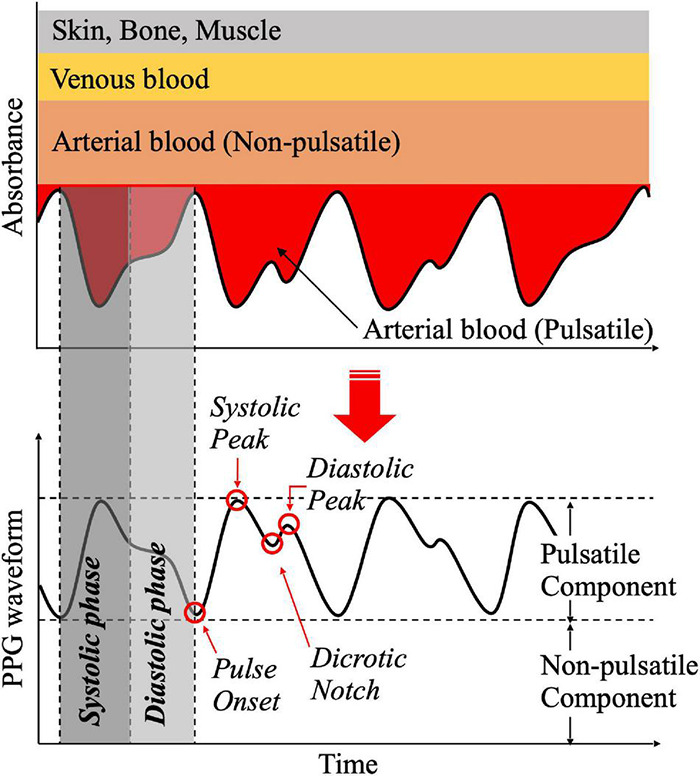
Principle of phototoplethysmogram generation and waveform features.

Figure 3 shows that PPG waveform is obtained from the amount of light absorption by inverting the light intensity recorded with a photodetector after the light is transmitted through or reflected from human tissue. In general, the PPG waveform is divided into a pulsatile component and a non-pulsatile component (Lee et al., 2011a). The pulsatile component, known as the alternating current (AC) component, is related to changes in blood volume in the artery. It is synchronized with the cardiac cycle and is related to vasodilation, vasomotor, and vascular tones (Nitzan et al., 2006; Shelley et al., 2006, 2014; Allen, 2007; Shelley, 2007; Reisner et al., 2008). It can be used to detect ventricular tachycardia and ventricular fibrillation (Alian and Shelley, 2014). The non-pulsatile component, known as the direct current (DC) component, refers to the remaining components excluding the pulsatile component of the PPG waveform (Challoner, 1979; Nilsson et al., 2003a,b). Non-pulsatile components are affected by biological characteristics, such as tissue composition and basic blood volume of the measurement site, as well as external factors, such as ambient light and measurement device specifications. It has been reported that respiration, vasomotor activity, Traube–Hering–Mayer wave, and thermoregulation can also affect the non-pulsatile component (Hertzman and Dillon, 1940; Hertzman and Roth, 1942; Senay et al., 1963; Allen and Murray, 2000a,b). The amplitude of the PPG waveform has an arbitrary unit, because the physical characteristics, such as oxygen-carrying capacity, bone size, skin color, blood vessel distribution, cardiac output, vascular stiffness, and vascular compliance, differ from person to person (Zhang et al., 2001; Krishnaswamy and Baranoski, 2004; Valencell, 2015). Its measurement depends on experimental environment, such as ambient light (Li et al., 2014; Xu et al., 2017).

The PPG waveform changes according to cardiac activity. It may also change because of respiration, autonomic nervous system activity, arterial activity, and venous activity (McKay et al., 2014; Pimentel et al., 2015; Bentham et al., 2018; Lakshmanan et al., 2018; Yuan et al., 2018). The PPG waveform includes cardiac activity and lung activity by frequency analysis. Shin and Min reported that most of the energy of the waveform is contained up to the 3rd harmonics (Shin and Min, 2017). The PPG waveform has a rising curve according to increase in capillary blood volume by cardiac contraction, and a descending curve according to decrease in capillary blood volume by cardiac dilation. It is repeated according to cardiac activity. At that time, rising curve is defined as the systolic phase of the PPG waveform, while descending curve is defined as the diastolic phase of the PPG waveform. Figure 3 shows the PPG waveform of one pulsation and various feature points. Pulse onset is defined as the point where pulsation begins at the point where blood volume is lowest before the systolic phase. Systolic peak is defined at the point where blood volume is maximized. Transient rising and falling of the PPG waveform during diastole occur when blood volume in capillaries temporarily increases again because of the occurrence of a pressure gradient in the opposite direction to the blood flow, just before the aortic valve closes (Dahlgren et al., 1991; He et al., 1995). At this time, recessed point is defined as a dicrotic notch, and the point at which the first derivative of the waveform is closest to zero after the systolic peak is defined as a diastolic peak (Millasseau et al., 2002). PPG waveform can change because of body composition, physiological status, and external stimuli. A previous study reported that it is difficult to use the absolute value of PPG amplitude for comparison, because it can change according to the characteristics of body tissues and individual characteristics, such as race, skin color, fingernail color, and finger size (Alian and Shelley, 2014). Moreover, PPG baseline is affected by respiration, vascular compliance, vascular tone, pain, and drug use (Nitzan et al., 2000; Shelley et al., 2006; Shelley, 2007). The amplitude of the systolic peak, a representative characteristic of the PPG waveform, has been reported to have a significant correlation with microvascular expansion, and is in proportion to the cardiac output (Dorlas and Nijboer, 1985; Murray and Foster, 1996). In addition, results from studies related to anesthesia, sympathetic activation, and use of vasoconstrictors related to autonomic nervous system activity have confirmed that when the peripheral vasculature is dilated, the amplitude of the systolic peak is increased, while when vasculature is constricted, it is decreased (Korhonen and Yli-Hankala, 2009). Dicotic notch changes with vascular tone and vascular compliance. It has been found that the location of notch occurrence is advanced at a high vascular tone (Shi et al., 2009). In addition, it has been reported that the time difference between diastolic peak and systole peak decreases with aging (Yousef et al., 2012).

### Photoplethysmogram Features and Clinical Applications

#### Basic Features Based on the Original Photoplethysmogram Waveform

**FIGURE 4:**
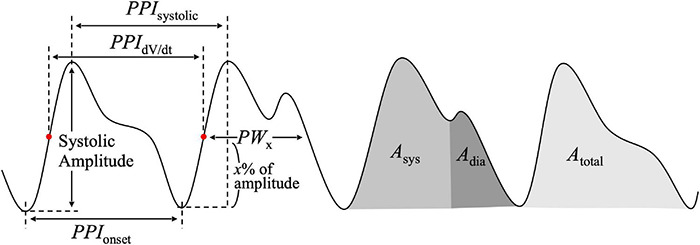
Features of the photoplethysmogram waveform. *PPI*_systolic_, interval between systolic peaks of adjacent pulse; *PPI*_dV/dt_, interval between maximum dV/dt of adjacent pulse; *PPI*_onset_, interval between pulse onsets of adjacent pulse; *PW*_x_, pulse width at *x*% of systolic amplitude; *A*_sys_, systolic area; *A*_dia_, diastolic area; *A*_total_, total pulse area.

Figure 4 shows the basic features obtained directly from the PPG waveform. Such PPG features are frequently used clinically (see Table 1). Systolic amplitude refers to the maximum amplitude of the PPG systolic phase. This is a feature related to the pulsatile component of blood volume (Asada et al., 2003). Systolic amplitude is highly correlated to stroke volume (Murray and Foster, 1996). It is directly proportional to the vasodilatation of the local body site where PPG is measured (Dorlas and Nijboer, 1985). A pulse width related index, PW_50_, refers to the pulse width between points corresponding to 50% of the PPG systolic peak amplitude, and shows a high correlation with systemic vascular resistance (Awad et al., 2007). Regarding pulse area, this is a feature that is calculated as the total area of the PPG waveform; it changes according to surgical skin incision (Seitsonen et al., 2005). Inflection point area ratio is calculated as the area ratio between the systolic and diastolic sections based on the dicrotic notch, and is correlated with total peripheral resistance (Wang et al., 2009). Pulse-to-pulse interval is obtained from the time interval between the characteristic points of two adjacent pulses of PPG. Pulse onset, systolic peak, and maximum value of derivative PPG are mainly used to measure pulse-to-pulse intervals. Pulse-to-pulse interval refers to one cycle of cardiac activity (Linder et al., 2006; Fu et al., 2008; Jubadi and Sahak, 2009; Gil et al., 2010). By calculating the pulse width ratio at different systolic amplitudes, the characteristic of an individual’s cardiovascular system by exercise could be determined (Poon et al., 2004). Pulse rate variability obtained through the pulse-to-pulse interval of PPG shows high correlation with the traditional heart rate variability obtained through electrocardiogram, and has been introduced as a surrogate method for measuring electrocardiogram-based heart rate variability under resting conditions (Lu et al., 2008). However, it was reported that PRV could be differ from HRV under dynamic conditions, such as exercise or mental stress conditions (Schäfer and Vagedes, 2013; Mejía-Mejía et al., 2020).

**TABLE 1:** Summary of photoplethysmogram (PPG) features and clinical relationship.

Feature type		Definition	Description	Clinical use
Basic		Systolic amplitude	• Maximum amplitude of the PPG systolic phase.	• Pulsatile component of blood volume (Asada et al., 2003; Chua and Heneghan, 2006) • Stroke volume (Murray and Foster, 1996) • Local vasodilatation (Dorlas and Nijboer, 1985)
		Pulse width	• The width of pulse. It usually represented as a time interval between the *x*% of the maximum systolic amplitude of PPG.	• Systemic vascular resistance (Awad et al., 2007; Lee et al., 2011c)
		Pulse Area	• The total area of the PPG in a pulsation. • The area of the systolic section, or the area of the diastolic section, divided based on the dicrotic notch.	• Surgical skin incision (Seitsonen et al., 2005) • Total peripheral resistance (Wang et al., 2009)
		Pulse-to-pulse interval	• The time interval between the maximum systolic amplitudes of two adjacent pulsations of PPG. • The time interval between the pulse onsets of two adjacent pulsations of PPG. • Time interval between the points of maximum derivative of two adjacent pulsations of PPG.	• Cardiac cycle (Linder et al., 2006; Fu et al., 2008; Jubadi and Sahak, 2009; Gil et al., 2010) • The systolic amplitude and pulse interval ratio reflect the individual’s cardiovascular system characteristics (Poon et al., 2004) • Heart (or Pulse) rate variability (Lu et al., 2008)
Combined		Perfusion index	• The ratio of the amplitude of the pulsatile component to the non-pulsatile component of PPG.	• Peripheral perfusion (Lima and Bakker, 2006; Hasanin et al., 2017; Chu et al., 2018)
		Large artery stiffness index	• Index calculated by dividing the subject’s height by the time interval between the systolic peak and the diastolic peak.	• Arterial stiffness (Millasseau et al., 2002, 2003; Yousef et al., 2012))
		PPG augmentation index	• The ratio of the systolic peak amplitude to the diastolic peak amplitude of a PPG. • The ratio of the difference between the systolic peak amplitude and diastolic peak amplitude to the diastolic peak amplitude of a PPG.	• Arterial stiffness (Takazawa et al., 1998; Brillante et al., 2008; Rubins et al., 2008)
		Pulse transit time	• Time difference between the specific features of PPGs measured at two different body sites.	• Cuffless blood pressure (Foo et al., 2006; Liu et al., 2018)
Derivative	1st	Crest time	• Time interval between the pulse onset and the first zero-crossing of the derivative PPG.	• Longer in vascular disease or hypertension patients (Hertzman, 1937; Dillon and Hertzman, 1941)
		ΔT	• Time difference between the first and the second zero-crossing points proceeding in the positive to negative value of PPG derivative.	• Time taken for the blood ejected from the heart to pass to the peripheral blood vessel (Alty et al., 2007)
	2nd	b/a	• Ratio of the amplitude of the early systolic negative peak to the amplitude of the early systolic positive peak of SDPTG.	• Proportional to the stiffness of blood vessels, and increases with age (Takazawa, 1993; Imanaga et al., 1998; Baek et al., 2007) • Inversely related to lead poisoning (Aiba et al., 1999) • Proportional to the Framingham risk score (Otsuka et al., 2006)
		c/a	• Ratio of the amplitude of the late systolic re-increasing peak to the amplitude of the early systolic positive peak of SDPTG.	• Vascular stiffness, and decreases with age (Takazawa, 1993; Baek et al., 2007) • Identifying hypertensive patients (Simek et al., 2005)
		d/a	• Ratio of the amplitude of the late systolic re-decreasing peak to the amplitude of the early systolic positive peak of SDPTG.	• Inversely proportional to vascular stiffness, and decreases with age (Takazawa, 1993; Baek et al., 2007) • Evaluation of vasoactive agents (Takazawa, 1993; Baek et al., 2007)
		e/a	• Ratio of the amplitude of the early diastolic positive peak to the amplitude of the early systolic positive peak of SDPTG.	• Inversely proportional to vascular stiffness, and decreases with age (Takazawa, 1993; Baek et al., 2007)
	(b-c-d-e)/a	• Ratio of the amplitude of all of the late systolic re-increasing peaks, the late systolic re-decreasing peak, and the early diastolic positive peak subtracted from the early systolic negative peak, to the amplitude of the early systolic positive peak of SDPTG.	• Vascular aging assessment (Takazawa, 1993; Baek et al., 2007) • Atherosclerosis assessment (Takazawa, 1993; Baek et al., 2007)
	(b-e)/a	• Ratio of the amplitude of the early diastolic positive peak subtracted from the early systolic negative peak, to the amplitude of the early systolic positive peak of SDPTG.	• Substitute indicator when c and d waveforms of indicator (b-c-d-e)/a are not identified (Takazawa, 1993; Baek et al., 2007)
	(b-c-d)/a	• Ratio of the amplitude of all of the late systolic re-increasing peaks and the late systolic re-decreasing peak subtracted from the early systolic negative peak, to the amplitude of the early systolic positive peak of SDPTG.	• Increases with chilly sensation (Ushiroyama, 2005)

*PPG, photoplethysmogram; SDPTG, second derivative PPG.*

#### Combined Features of Photoplethysmogram

Features that combine several characteristic points of PPG include perfusion index, large artery stiffness index, PPG augmentation index, and PTT. Perfusion index is calculated as the ratio of the pulsatile component to the non-pulsatile component of the PPG. It is used as an index to evaluate peripheral perfusion (Lima and Bakker, 2006; Hasanin et al., 2017; Chu et al., 2018). Aortic stiffness index is calculated by dividing the height of a subject by the time interval of the maximum amplitude of the systolic and diastolic peaks. It represents the stiffness of an artery (Millasseau et al., 2002, 2003; Yousef et al., 2012)). PPG augmentation index is used as a feature for the stiffness of arterial vessels; it is calculated as the ratio of the amplitude of the systolic peak to the amplitude of the diastolic peak (Takazawa et al., 1998; Brillante et al., 2008) or by dividing the difference between the amplitude of the systolic and diastolic peaks by the amplitude of the systolic peak (Rubins et al., 2008). PTT is obtained through the time difference between specific feature points of PPGs measured in two different body sites. It is used as a feature to estimate blood pressure (Foo et al., 2006; Liu et al., 2018). Table 1 describes the common features.

#### Derivative Features of Photoplethysmogram

**FIGURE 5:**
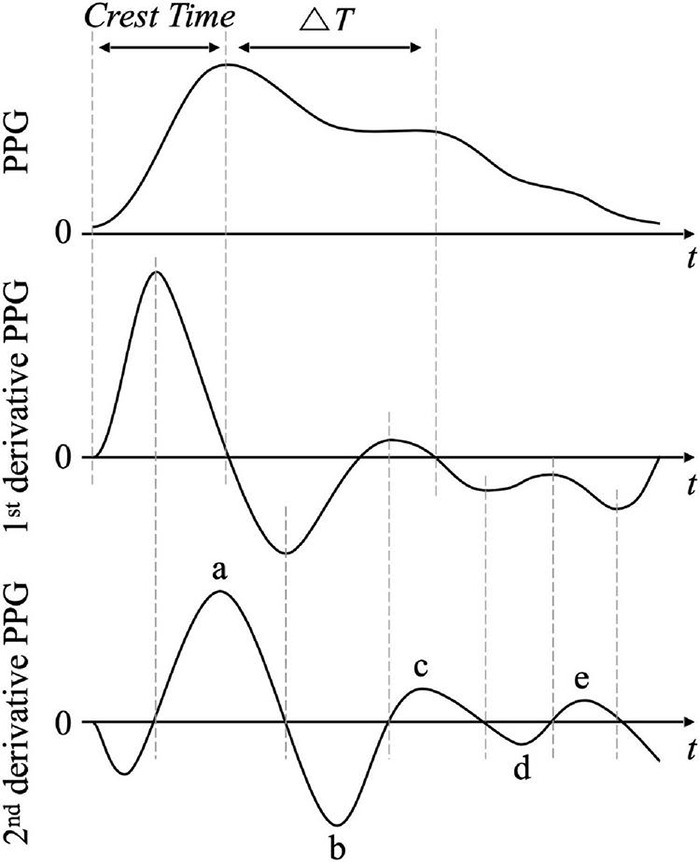
Waveform and features of photoplethysmogram (PPG, top), derivative PPG (middle), and second derivative PPG (bottom). Crest time is the elapsed time from pulse onset to systolic peak. Δ*T* is the time interval between systolic peak and diastolic peak that is defined by the second downward zero-crossing time in derivative PPG. In the second derivative PPG, a, b, c, d, and e are the early systolic positive peak, early systolic negative peak, late systolic re-increasing peak, late systolic re-decreasing peak, and early diastolic positive peak, respectively.

Since the 1970s, studies have shown that the differential waveform of PPG has physiological significance. After Takazawa et al. (1998) showed a correlation between the second derivative PPG and aging, PPG derivative studies began to receive full-scale attention. Figure 5 shows a PPG waveform, derivative PPG, and second derivative PPG. Derivative and second derivative PPGs are advantageous for representing spatiotemporal variations of PPG with respect to peak position, inflection point, number of peaks, ascending slope, and descending slope. They can be used as an alternative method to detect dicrotic and diastolic peaks that are difficult to detect in original PPG waveforms. The first-order derivative waveform of PPG is also called velocity plethysmography (VPG). The first derivative waveform of PPG can be used to extract crest time, the time taken to contract from the pulse onset of the original signal to the systolic peak, or time interval ΔT from the systolic peak to the diastolic peak. Crest time can be defined as the time taken from the start point of the VPG waveform to the following zero-crossing. Hertzman (1937) and Dillon and Hertzman (1941) proposed that crest time could be longer in patients with vascular disease or hypertension than in a normal group. Alty et al. (2007) reported that among the features extracted from the first derivative PPG, ΔT, defined as the time difference between the first and second zero-crossing points proceeding in the positive to negative value in the VPG waveform and crest time shows high accuracy for predicting cardiovascular disease. They showed that ΔT is related to the time it takes for blood ejected from the heart to pass to peripheral blood vessels, and that it can classify cardiovascular diseases with an accuracy of 87.5% using a support vector machine. The second-order derivative PPG waveform is also called the second derivative of phothoplethysmogram (SDPTG), second derivative of the digital volume pulse (SDDVP), and acceleration plethysmogram (APG). Takazawa et al. (1998) defined the peaks and valleys of the second-order differentiated PPG waveform as *a*, *b*, *c*, *d*, and *e*, as shown in Figure 5. They showed that combined indices, such as *b*/*a*, *c*/*a*, *d*/*a*, and *e/a*, had a significant correlation with aging.

#### Other Clinical Applications

In addition, studies for predicting various parameters or diagnosing diseases have been conducted using PPG. In addition to basic heart rate estimation, PPG is used for blood pressure estimation (Poon and Zhang, 2005; Muehlsteff et al., 2006; He et al., 2014; Nabeel et al., 2017; Wang et al., 2018; El Hajj and Kyriacou, 2020), vascular aging assessment (Takazawa et al., 1998; Bortolotto et al., 2000; Millasseau et al., 2003; Baek et al., 2007; Jubadi and Sahak, 2009; Wang et al., 2009; Yousef et al., 2012; Dall’Olio et al., 2020; Korkalainen et al., 2020), arterial fibrillation prediction (Poh et al., 2018; Kwon et al., 2019; Aschbacher et al., 2020; Cheng et al., 2020; Pereira et al., 2020), diabetes prediction (Shan et al., 2016; Tang et al., 2017; Poh et al., 2018; Eerikäinen et al., 2019; Guo et al., 2019; Kwon et al., 2019; Proesmans et al., 2019; Yang et al., 2019; Aschbacher et al., 2020; Cheng et al., 2020; Pereira et al., 2020), peripheral vascular disease assessment (Allen and Murray, 1993; Alnaeb et al., 2007; Bentham et al., 2018; Allen et al., 2021), surgical and postoperative pain assessment (Ahonen et al., 2007; Struys et al., 2007; Kallio et al., 2008; Hasanin et al., 2017; Yang et al., 2018; Seok et al., 2019), heterogeneous bio-signal (e.g., ECG) reconstruction (Zhu et al., 2021), hemodynamic parameter estimation such as cardiac output (McCombie et al., 2005; Wang et al., 2009, Wang et al., 2010, 2014; Lee et al., 2013) or stroke volume (Liu et al., 2020a,b), sleep monitoring including apnea and hypopnea detection (Behar et al., 2014; Uçar et al., 2015; Park and Choi, 2019; Hilmisson et al., 2020; Lazazzera et al., 2020), and emotional recognition (Rakshit et al., 2016; Ayata et al., 2018; Goshvarpour and Goshvarpour, 2018, 2020; Lee et al., 2019).

### Photoplethysmogram Noise

**FIGURE 6:**
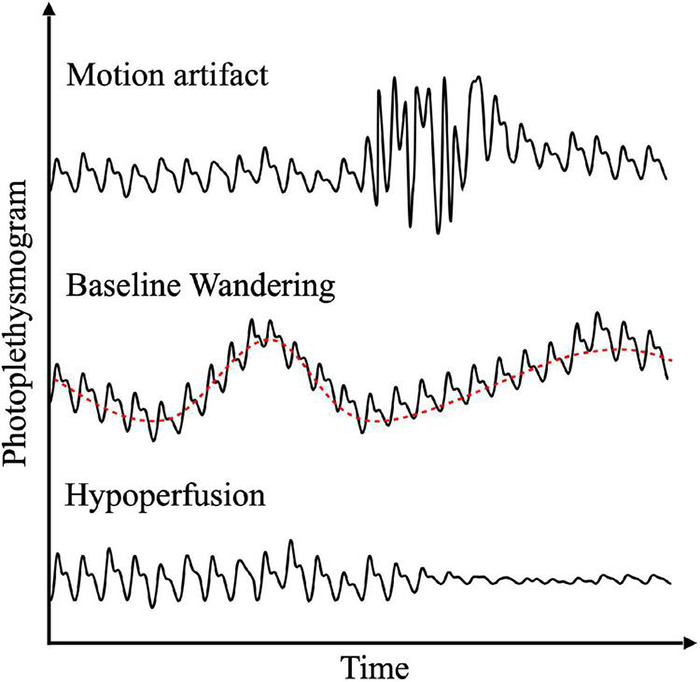
Examples of representative PPG distortion due to motion artifact, baseline wandering, and hypoperfusion (from top to bottom).

The results of our literature research related to PPG noise reduction are summarized. Representative noises that affect PPG analysis results include MAs related to body movement and sensor attachment, baseline change due to respiration and body movement, and hypoperfusion due to decreased peripheral perfusion. Figure 6 describes these representative photoplethysmogram distortions. Each noise is described in the following subsections.

#### Motion Artifact

Motion artifact, which is mainly caused by body motions, such as hand movement, walking, and running, is a critical noise when measuring PPG. Depending on probe type and light source, PPG measurement may be more sensitive to MA; it has been reported that red and green wavelengths are more robust to MA (Matsumura et al., 2020). In addition, depending on measuring sites, it was shown that MA in ear PPG is less than in finger or forehead PPG (Selvaraj et al., 2011). Since MA is known to have a frequency range of 0.01–10 Hz, the major component of PPG can be distorted by overlapping with the main frequency band (0.5– 5 Hz) of PPG (Bagha and Shaw, 2011; Rojano and Isaza, 2016; Lee et al., 2020). Such distortion makes it difficult to detect important features during analysis, and that causes false diagnosis. Therefore, an MA must be removed or corrected prior to analysis. In MA removal using a frequency domain filter, a high-pass filter is mainly used. Joseph et al. reported that a high-pass filter with a cut-off frequency of 0.15 Hz does not change pulse shape, while maintaining an ideal ratio of the pulsatile and non-pulsatile components of PPG (Joseph et al., 2014). In addition, a study by Allen and Murray comparing the performance of a total of 90 filters by combining 9 filter types and 10 filter orders found the fourth-order Chebyshev type II filter to have the best performance in improving PPG signal quality (Allen and Murray, 2004). However, since the frequency domain filter alone has limitations in removing MA according to various motion intensities and motion types, studies on removal of MAs are being conducted using algorithms based on filters, accelerometers, and multiple wavelengths. The MA reduction method by independent component analysis (ICA) is a method of removing only the MA component by extracting independent components corresponding to PPG and MA from PPG containing MAs, assuming that PPG and MA are random vectors. Kim and Yoo (2006) qualitatively confirmed that the PPG and MA components can be separated by ICA. Lee et al. (2020) proposed a method to reduce MA by applying ICA to the multi-channel PPG obtained with a multi-wavelength light source. In their study, the MA included in PPG during walking, fast walking, and running was reduced by ICA. PPG peak was then detected. The position of the detected PPG peak was 99, 96.2, and 82.0%, consistent with the QRS position of the ECG in walking, fast walking, and running, respectively. In MA removal using adaptive noise cancelation (ANC), the accelerometer and PPG are measured simultaneously. The damaged part of the PPG is found and removed by the motion detected by the accelerometer (Widrow et al., 1975). Foo and Wilson (2006) used ANC to remove the MA generated from light motion, while Poh et al. (2010) used ANC to remove the MA generated from walking and running. In addition, Han et al. (2007) proposed a method of removing MA by simultaneously measuring PPG and acceleration, and applying a two-dimensional active noise cancelation algorithm. This algorithm can reduce the signal distortion rate from 52.3 to 3.53 at a frequency of 1–2.5 Hz using the 4th-order normalized least mean square (NLMS) adaptive filter. Seyedtabaii and Seyedtabaii (2008) proposed that adaptive filtering based on the Kalman filter may be effectively used for MA reduction. A study by Reddy et al. (2008) reduced the normalized root mean squared error by 35 dB after eliminating MA components in a frequency domain by cycle-by-cycle Fourier series analysis for each pulse in PPG. Patterson and Yang (2011) showed that MA for vertical finger movement and rotational movement could be removed through stationary wavelet transform. The error with HR and HRV obtained from ECG can be reduced (Joseph et al., 2014).

#### Baseline Wandering

The baseline of the pulsatile component of PPG and AC amplitude of PPG can be changed by various factors, such as respiration, sympathetic nervous system activities, and thermoregulation (Allen, 2007). The change in PPG baseline interferes with the analysis of the AC component of PPG. Therefore, to accurately analyze the AC component of PPG, Timimi et al. (2017) proposed a method of directly removing the change in baseline and a method of removing the change in baseline by subtraction from the measured signal based on estimation of the change in baseline. Jang et al. (2014) reported that high-pass filtering (HPF) is frequently performed in the method of directly removing the baseline. The frequency component of the AC of PPG is a component related to pulsation. This is normally higher than 0.5 Hz (30 bpm) in a healthy person. However, the respiratory component that causes baseline change has a frequency range of 0.15–0.5 Hz. HPF is performed to remove baseline movement located in the low-frequency range without damaging the AC component, based on the frequency range difference of signals. HPF is simpler and more convenient to performed than the method of baseline removal based on direct estimation. However, when the frequency component of PPG AC is lower than the cut-off frequency band of HPF, this method may cause signal distortion. As a method of indirectly estimating and removing the baseline, interpolation methods, such as linear interpolation and cubic spline interpolation, can be used for baseline estimation. A method combining wavelet and least mean square (LMS) adaptive filter can also be used (Wang et al., 2003). The linear interpolation method can simply estimate the baseline with a low-order polynomial. However, linear interpolation has the disadvantage that it is not very precise and the interpolant is not differentiable. Cubic spline interpolation can compensate for this discontinuity of signal by estimating the change in baseline through a cubic polynomial. In baseline removal using the interpolation technique, baseline wander is removed by subtracting the estimated baseline from PPG. In the method of removing baseline variation by combining wavelet transformation and LMS adaptive filter, the baseline component extracted by wavelet transformation is applied to the LMS adaptive filter to remove the baseline component. Then, the PPG from which the baseline is removed is obtained through inverse wavelet transform. Considering that PPG has non-stationary characteristics, wavelet-based baseline estimation may be appropriate. However, since both wavelet and adaptive filtering must be performed, its computational complexity may be high compared to other methods. In addition, due to the transition band of the filter, such method is unsuitable for cases with short signals.

#### Hypoperfusion

Hypovolemia, hypothermia, vasoconstriction, and decreased cardiac output or mean arterial pressure may weaken changes of blood volume in blood vessels, called poor perfusion or low perfusion (Alnaeb et al., 2007). Hypoperfusion becomes more pronounced toward the peripheries of the body. It affects the pulsatile component of PPG, thus weakening amplitude change (Kyriacou et al., 2002). To improve the low perfusion waveform, Foo and Wilson applied a non-causal Wiener filter with a 0.1- to 15-Hz pass band (Foo and Wilson, 2006); they showed that the heart rate error estimated from low-perfusion PPG could be reduced to less than 5.12%. Shafique et al. (2012) proposed a method to improve low perfusion by simultaneously measuring PPG using a transmission-type and a reflection-type measuring device, and reconstructing the PPG using a summing amplifier. The reconstituted PPG showed higher sensitivity than single-mode PPG in PPG measurement in a low perfusion state that was forcibly generated using the cuff. Oxygen saturation measurement also showed lower failure rate than commercial products.

In addition to the movement, respiration, and low perfusion of a subject, there are numerous factors that can distort the PPG waveform. Typical examples include ambient light, temperature of the measuring site, skin pigmentation in the measurement body site, alignment of light source and photodetector, method of attaching the sensor to the skin, contact pressure between the sensor and the skin, and posture of a subject (Reynolds et al., 1991; Adler et al., 1998; Teng and Zhang, 2006; Zhang and Zhang, 2006; Lee et al., 2011b; Kim et al., 2016). Ambient noise reduction is mainly attempted through hardware improvement. Kim et al. (2016) developed a PPG readout chip equipped with a technique that can remove the effect of ambient light through a charge redistribution method after cross-sampling PPG mixed with ambient light with complementary metal–oxide–semiconductor (CMOS) process. Cold site temperature causes vasoconstriction of the measurement site and reduces perfusion, thereby degrading the quality of the measured signal (Khan et al., 2016). Massage or warming is known to be effective for increasing blood flow or perfusion, and improvement of signal quality through this method has also been reported (Bohusch et al., 1994; Foo, 2007; Freckmann et al., 2012). It is known through several studies that the signal-to-noise ratio of PPG measured according to skin color or pigmentation shows a significant difference (Fallow et al., 2013; Yan et al., 2017; Sañudo et al., 2019). Yan et al. (2017) suggested multi-wavelength measurement to be effective as a method to reduce deviation by skin type. In addition, Fallow et al. (2013) reported that high-resolution PPG can be obtained for various skin types with green wavelength under resting conditions, and green or blue wavelength under exercise conditions. When measuring PPG, probe pressure causes change in PPG waveform and could affect analysis result (Dresher and Mendelson, 2006b; Liu et al., 2015). A method of adjusting the contact force by optimizing the housing design of the PPG probe (Dresher and Mendelson, 2006a) or using a measuring platform with a built-in force regulator in the probe (Sim et al., 2018) has been proposed as a method to improve non-uniform contact force.

### Photoplethysmogram Signal Processing

#### Photoplethysmogram Preprocessing

Table 2 summarizes the pre-processing techniques of PPG. Because of the simplicity of its waveform, PPG has a relatively simple pre-processing process. Our literature search found that most PPG pre-processing was dependent on frequency filtering to remove high-frequency or low-frequency noise. In frequency filtering, the lower bound of the passband in most studies is about 0.5 Hz (Sukor et al., 2011; Papini et al., 2018; Canac et al., 2019; Pradhan et al., 2019) to remove the DC component below 0.1 Hz and respiratory component in the 0.1–0.5 Hz band while obtaining only the AC component of PPG. The upper bound of the bandpass filter is usually determined considering that the main frequency components of PPG are included within the fourth harmonics in the frequency domain. The upper bound of the frequency filter at 10 Hz as the position of the fourth harmonics is often used when heart rate is 150 bpm (2.5 Hz). Thus, in the general case, the low pass filter that has a 10-Hz cutoff frequency can include most PPG frequencies (Papini et al., 2018; Canac et al., 2019; Liu et al., 2020a,b). The Butterworth, Chebychev I, and finite impulse response (FIR) filters are mainly used for frequency filtering. PPG pre-processing is also performed by decomposing the waveform into several frequency components, removing noise for each component, and then recombining them. A representative of these methods is the method based on empirical mode decomposition (EMD) or wavelet decomposition. With the EMD-based method, the noise component is removed by excluding the intrinsic mode function (IMF) based on a specific frequency after obtaining the IMF of PPG and then recombining it. Lu et al. (2008) removed low-frequency noise and the trend of PPG by recombining only IMF with a dominant frequency > 0.5 Hz. Similarly, in a study using a wavelet transform, noise is removed by obtaining a sub-band signal through wavelet decomposition and combining specific sub-bands. Vadrevu and Manikandan (2018) showed that low-frequency and high-frequency noise of PPG can be effectively removed in the preprocessing step to detect the peak by recombining the sub-band signal after applying the stationary wavelet transform. Shin et al. (2010) used the discrete cosine transform to remove noise outside the 0.5- to 10-Hz band, and found that it can be used for PPG pre-processing with sparse frequency characteristics. Selvaraj et al. (2011) showed that a high-order polynomial can be used to handle non-stationary dynamics. In addition to noise reduction, pre-processing is also performed for signal enhancement purposes. Kim et al. (2019) proposed an amplitude regularization technique using an envelope curve to reduce the fluctuation of PPG amplitude. Canac et al. (2019) used a moving differentiation filter to sharpen PPG upslope and eliminate high-frequency noise.

**TABLE 2:** Summary of preprocessing methods for PPG.

Preprocessing method	Details	Purpose
Frequency filtering	Bandpass filter	Reduction for high-frequency noise, baseline movement reduction
	- 1st order Butterworth [(0.5 – 5) Hz] (Sukor et al., 2011)	
	- 2nd order Butterworth [(0.2 – 10) Hz] (Liu et al., 2020b)	
	- 3rd order Butterworth [(0.4 – 10) Hz] (Papini et al., 2018)	
	- 4th order Butterworth [(0.5 – 50) Hz] (Pradhan et al., 2019)	
	- 4th Chebychev I [(0.5 – 16) Hz] (Ferro et al., 2015)	
	- 4th order Butterworth [(0.5 – 10) Hz] (Canac et al., 2019)	
	- 64th order FIR [(0.1 – 10) Hz] (Selvaraj et al., 2011)	
	- Discrete cosine transform filtering [(0.5 – 10) Hz] (Shin et al., 2010) High pass filter	
	- 4th order Butterworth, cut-off: 0.01 Hz (Fischer et al., 2017) Low pass filter	
	- 2nd order Butterworth, cut-off 10 Hz (Liu et al., 2020a)	
	- 4th order Butterworth, cut-off 15 Hz (Fischer et al., 2017)	
Empirical mode decomposition	Waveform reconstruction using intrinsic mode functions whose dominent frequency is > 0.5 Hz (Lu et al., 2008)	Reduction for low-frequency (<0.5 Hz) noise and baseline noise reduction
Wavelet transform	Signal reconstruction using specific sub-bands after stationary wavelet transform (Vadrevu and Manikandan, 2018)	Suppression of background artifacts and noises
Independent component analysis	Reducing motion artifact using frequency domain independent component analysis based on red and infrared signal (Krishnan et al., 2008)	Motion artifacts reduction
Moving difference filter	Calculating the difference with the sample after a window size of a moving window (Canac et al., 2019)	Enhancing upslope of the photoplethysmogram
Curve fitting	Amplitude normalization	Eliminating non-stationary dynamics
	- Amplitude compensation curve (Kim et al., 2019)	
	Detrending	
	- 32nd-order polynomial fitting (Selvaraj et al., 2011)	

#### Photoplethysmogram Peak Detection

Peak detection is essential for analyzing PPG. Detection methods based on zero-crossing, local maxima or minima (LCM), adaptive threshold, and machine learning have been proposed. Zero-crossing is a method that can find the point where the sign of the slope changes, in the same way as a quasi-periodic signal peak detection method. However, since the zero-crossing-based method is highly likely to erroneously detect peaks due to tiny fluctuations of signals, various filtering methods must be applied in the peak detection method based on zero-crossing to simplify the PPG waveform. To detect PPG peaks using the zero-crossing method, Canac et al. (2019) used a 0.5- to 10-Hz 4th order Butterworth filter and a moving difference filter for the detection of pulsating wave peaks, while Kavsaoğlu et al. (2016) segmented the PPG signal and divided it by the sign of each slope. In addition, a method for detecting peaks through zero-crossing after wavelet or Hilbert transform of the PPG signal has been reported (Scholkmann et al., 2012; Ferro et al., 2015). Ferro et al. (2015) detected onsets and systolic peaks of a pulse wave with low complexity and low computation cost; however, their study was validated with only a small number of study subjects (*N* = 10) in a noise-free environment. Vadrevu and Manikandan (2018) decomposed the PPG component through wavelet decomposition and then detected systolic and onset peaks, without by knowledge rule-based post-processing. This approach showed over 99% of sensitivity and predictivity on the total number of 116,255 beats taken from three PPG databases; however, it has relatively high complexity from the use of wavelet decomposition. Also, it is hard to apply for real-time application because of being designed for a non-causal system. One of the most frequently used PPG peak detection methods is a method based on LCM detection. LCM is a method of finding the maximum or minimum value within a specific region based on a pre-defined threshold. In LCM peak detection, peaks are detected by repeated window sliding and peak detection. The detection threshold can have a fixed value or an adaptive value. Lu et al. (2008) determined the threshold at a certain ratio of the maximum PPG value. Xu et al. (2008) set the threshold based on pulse height and detected peaks by comparing the heights of candidate peaks within a 2-s window. The LCM method requires appropriate window size selection. It has the disadvantage that if there are large-scale baseline changes due to respiration or other various causes, accurate detection becomes difficult. Shin et al. (2009) proposed an adaptive threshold PPG peak detection method based on a dynamic threshold that tracks the signal amplitude and finds peaks at the maximum amplitude outside the refractory period. Adaptive threshold is known to overcome the shortcoming of the LCM method that is vulnerable to baseline noise, such as baseline fluctuations due to respiration, with better detection performance than the LCM method for detecting the peaks of PPG signals. Scholkmann et al. (2012) proposed a method of estimating the local maxima by obtaining a scalogram of wavelet transformation and then rescaling it. In this method, the local maxima scalogram was first calculated and rescaled. Peaks were then detected by row-wise summation and column-wise standard deviation. The proposed method has high robustness against high-frequency and low-frequency noise, and has a potential to be used for detection of various signal peaks. Recently, there have been attempts to apply deep learning to PPG peak detection. Orjuela-Cañón et al. (2013) proposed a PPG peak detection method based on a self-organized map. Although the PPG peak detection method using machine learning has not yet been confirmed to have stable performance, performance improvement is expected in the future through continuous development. The PPG peak detection methods mentioned above can detect peaks with high accuracy in PPG signals without noise. However, their PPG peak detection performance may be greatly degraded because of MAs, baseline wandering, and low perfusion. In addition, in the case of systolic peak, there is the possibility of erroneous detection due to interference of the dicrotic and diastolic peaks. Therefore, noise removal through proper signal pre-processing and restoration of distorted signals remains important for PPG utilization. Table 3 briefly summarizes the PPG peak detection techniques.

**TABLE 3:** Overview of studies on peak detection of PPG.

Study	Subjects (age)	Recording time (minute)	Experimental condition (default is resting)	Device used	Sensor position	Peak type	Results
Canac et al., 2019	108 patients (30–64)	n.s.	Supine	Multi-Dop X (Compumedics DWL, Singen, Germany)	Head	Onset	Acc: 99.5%
Kavsaoğlu et al., 2016	20 healthy adults (18–41)	1	Sitting	SDPPG_V2.0 (APMKorea, Daejeon, Korea)	n.s.	Systolic	Acc: 100%
Ferro et al., 2015	10 healthy adults (19.3 ± 1.4)	5	Supine	In-house device	n.s.	Onset, systolic	Acc: 95% (onset) Acc: 100% (systolic)
Vadrevu and Manikandan, 2018	20 healthy adults (18–35)	10–15	Sitting	In-house sensor	Finger	Onset, systolic	Acc: 99.3% (onset) Acc: 99.3% (systolic)
Lu et al., 2008	10 healthy adults (26 ± 7.5)	20	Upright, supine	MP506 (Medtronic, MN, United States)	n.s.	Onset	Obtaining pulse rate variability highly correlated with heart rate variability
Shin et al., 2009	18 healthy adults (17–30)	5	Supine (respiratory control), Sitting (spontaneous breathing)	PPG 100C (Biopac, CA, United States)	Finger	Onset, systolic	Acc: 98.9% (onset) Acc: 98.2% (systolic)
Scholkmann et al., 2012	n.s.	3.5	n.s.	Functional near-infrared spectroscopy MCP-II (n.s.)	Prefrontal cortex	Systolic	Acc: 100%
Orjuela-Cañón et al., 2013	7 healthy adults (19.3 ± 1.5)	5	Supine	n.s.	n.s.	Onset, systolic	Acc: 100% (onset, systolic)

*Acc, accuracy; n.s., not specified.*

#### Photoplethysmogram Waveform Reconstruction

**FIGURE 7:**
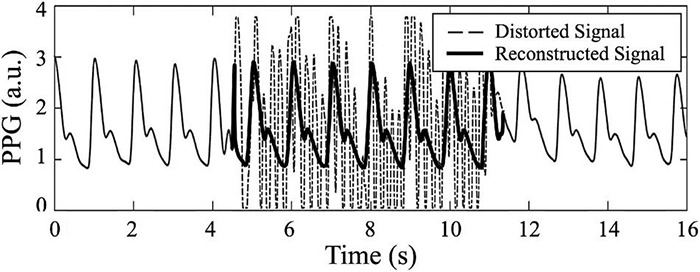
Example of PPG waveform reconstruction. Dashed line is distorted PPG, while bold line is reconstructed PPG.

Photoplethysmography (PPG) waveform reconstruction is mainly performed to restore the damage to PPG caused by noise, such as MAs. If distortion of PPG is not severe with preservation of the main components of the waveform, PPG can be decomposed into wavelet components through discrete wavelet transform, and noise can be removed for each component to restore PPG (Tang et al., 2016). In addition to the reconstruction method in the time–frequency domain using discrete wavelet transform, a method of reconstructing PPG using eigen-decomposition has also been proposed (Salehizadeh et al., 2014). In this method, after eigen-decomposition is performed to extract the eigen components of PPG, PPG is restored only with the main components from which the noise components are removed. When most of the waveform information is lost because of severe distortion of PPG, detecting the damaged part and estimating the waveform of the corresponding part to restore it using a machine learning technique, such as recurrent neural network, have been reported (Tarvirdizadeh et al., 2018; Roy et al., 2019). In addition to restoring distorted parts, reconstruction of the PPG waveform can be performed to enhance the waveform. To equalize PPG amplitude fluctuations when severe fluctuations in the PPG baseline or amplitude occur, Kim et al. (2019) proposed a method of compensating the PPG amplitude using an amplitude compensation curve generated from the envelope of the PPG waveform. Figure 7 shows an example of PPG waveform reconstruction.

### Signal Quality Index

#### Feature-Based Signal Quality Assessment

**FIGURE 8:**
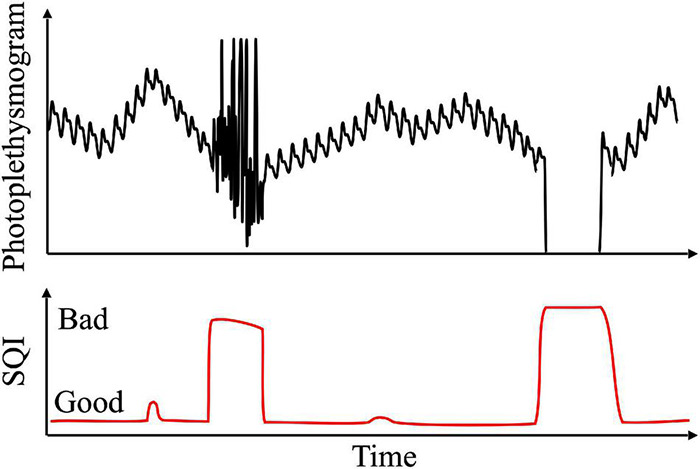
Example of signal quality assessment using signal quality index (SQI).

Signal quality index (SQI) is generally used to evaluate signal quality, such as signal-to-noise ratio. It is applied before signal analysis to evaluate the usability of a signal (see Figure 8). Pulse quality index refers to the quality of pulses constituting the signal, and is used to evaluate the quality of the pulse waveform as part of the SQI. Waveform quality is the most important factor in deriving accurate analysis results. A signal of low quality increases the false alarm, as well as probability of occurrence of an analysis error, which can lead to clinical misdiagnosis. For example, if part of the waveform is lost when calculating heart rate, an error may occur in peak detection, resulting in change in heart rate.

Such a case may also occur in a patient-monitoring device that monitors the physiological signals of a patient in real time. Patient monitoring alerts medical staff when vital signs, such as HR, deteriorate. As in the previous case, false alarms may occur because of deterioration of waveform quality caused by movement or sensor attachment condition, although the condition of a patient is normal. These false alarms cause noise stress to medical staff and can interfere with their accurate and immediate response. In addition, because of recent increase in the use of PPG in the field of mobile healthcare, there is an increasing demand to improve its usability in the mobile environment. However, PPG is very vulnerable to MAs. It may suffer from signal quality degradation due to various factors, such as low perfusion or ambient light. Therefore, it is very important to distinguish between analyzable and non-analyzable sections of the measured PPG signals. To improve the accuracy of analysis results, SQI evaluation of PPG is becoming more important, especially in the mobile environment.

Table 4 summarizes previous studies on signal quality assessment. Among methods for evaluating SQI, the rule-based method can determine the availability of a signal by sequentially determining various parameter values, such as amplitude, beat interval, and feature value, describing the PPG waveform based on specific thresholds. Fischer et al. (2017) evaluated signal quality using thresholds for amplitude, rise time, pulse-to-pulse interval, number of diastolic peaks, and waveforms of a pulsation. Sukor et al. (2011) similarly proposed a method for evaluating SQI by applying a decision tree to amplitude, beat interval, waveform width, ensemble mean of all beats, and Euclidean distance; the quality of PPG was distinguished into three grades and was evaluated with an accuracy of 83 ± 11% and sensitivity of 89 ± 10% compared to the expert-labeled gold standard.

**TABLE 4:** Overview of studies on PPG signal quality assessment.

Study	Number of subjects (age)	Recording time (minute)	Experimental condition (default is resting)	Device used	Sensor position	Classification grades	Results
Fischer et al., 2017	69 unspecified (>18)	30	n.s.	n.s.	n.s.	2	PPV: 98.6% Sen: 99.5% Acc: 98.4% Spe: 91.6% F1 score: 99.1%
Sukor et al., 2011	13 healthy adults (28 ± 4)	1	Sitting (movement)	n.s.	Finger	3	Sen: 89 ± 10% Acc: 83 ± 11% Spe: 77 ± 19%
Selvaraj et al., 2011	24 healthy adults (n.s.)	5–20	Supine (involuntary movement, 10 subjects), sitting (voluntary finger movement, 14 subjects)	MLT1020 (ADI Instruments, CO, United States), PPG 100 (Biopac, CA, United States)	Finger, ear, forehead	2	In involuntary movement, Acc: 99.0% (ear) Acc: 94.8% (finger) Acc: 93.3% (forehead) In voluntary movement (finger), Sen: 85.0% Spe: 99.4%
Elgendi, 2016	40 healthy adults (34.7 ± 6.6)	80 s	Exercise (movement)	Salus APG (Kashima Mediabind Co., Osaka, Japan)	Finger	3	F1 score Excellent: 86% Acceptable: 87.2% Unfit: 79.1%
Orphanidou et al., 2014	19 healthy adults (n.s.)	5	n.s.	EQ-02 Life Monitor (Hidalgo, Swavesey, United Kingdom), Wrist Ox2 3150 (Nonin Medical Inc., Plymouth, MN, United States)	Finger	2	Sen: 91% Spe: 95%
Li and Clifford, 2012	104 patients from MIMIC II database (n.s.)	n.s.	n.s.	n.s.	n.s.	3	Acc: 88.1% (training) Acc: 91.8% (testing)
Papini et al., 2018	16 healthy adults, 16 arrhythmia patients (n.s.)	Overnight or 24 h	Supine	n.s.	Finger	2	PPV: 97% (healthy) PPV: 95% (arrhythmia)
Karlen et al., 2012	Unspecified patients from Capnobase and Complex System Laboratory database (1–74)	2–8	n.s.	n.s.	n.s.	0–100	PPV: 99.2% Sen: 96.2%
Liu et al., 2020b	10 healthy adults (23.5 ± 1.7)	3	n.s.	CS2000 (medis, Ilmenau, Germany)	Neck (carotid artery)	3	In grade ‘high’, Sen: 81% Spe: 90% In grade ‘low’, Sen: 84% Spe: 93%
Liu et al., 2020a	14 healthy adults (22.7 ± 2.1)	3	n.s.	CS2000 (medis, Ilmenau, Germany)	Neck (carotid artery)	3	Acc: 89.5% (VGG-19) Acc: 92.5% (ResNet-50)
Naeini et al., 2019	n.s. (n.s.)	5 days	Ordinary life	E4 (Empatica, MA, United States), PulseOn (pulseon, Espoo, Finland)	Wrist	2	In grade ‘unreliable’, PPV: 76.74% Sen: 83.54% In grade ‘reliable’, PPV: 88.50% Sen: 83.33%
Pradhan et al., 2019	26 healthy adults (approx. 65)	24 h	Ordinary life	E4 (Empatica, MA, United States)	Wrist	5	Acc: 74.5%

*PPV, positive predictive value; Sen, sensitivity; Acc, accuracy; Spe, specificity; n.s., not specified.*

Orphanidou et al. (2014) evaluated the quality of PPG signal in two grades using heart rate, PPI interval and ratio, and template matching, resulting in sensitivity and specificity of more than 90%. Skewness and kurtosis are also frequently used features for signal quality evaluation. Krishnan et al. (2008) evaluated signal quality based on skewness and kurtosis. Selvaraj et al. (2011) evaluated signal quality in two grades through kurtosis and Shannon entropy. Elgendi (2016) classified PPG into three grades (excellent, acceptable, and unfit), and compared the performance of SQI evaluation indices, such as perfusion index, kurtosis, skewness, relative power, non-stationarity, zero-crossing, and entropy. Kurtosis and skewness are statistical measures for quantifying the characteristics of a normal distribution. Morphologically, “How sharp is the shape?” and “In which direction and by how much is the shape skewed?” can be quantified. From this morphological point of view, the kurtosis and skewness of the PPG pulse can comprehensively reflect the amplitude or position of the PPG shape, such as pulse width, systolic peak, diastolic peak, and dicrotic notch. In addition, since the kurtosis or skewness of the PPG pulse can be clearly distinguished from motion noise, this can be an effective means of distinguishing the PPG waveform from noise. On the other hand, kurtosis and skewness may be inefficient to detect the distortion caused by amplitude or pulse width in an abnormal range, because they are determined by relative shape, not by absolute value. The ratio of AC component to DC component of PPG is called perfusion index (PI), and it has been used in several studies to evaluate signal quality (Hartmut Gehring et al., 2002; Cannesson et al., 2008a). PI is useful in detecting waveform degradation by low perfusion, because it is known to reflect vasomotor tone that may affect the pulsatile absorption component; moreover, it is a direct indicator of low perfusion by itself.

Song et al. (2019) proposed PQR as a method for evaluating signal quality through high-frequency noise effect (P), baseline effect (Q), and MA effect (R). In the PQR method, a PQI score called rSQI is calculated by adding each score of P, Q, and R, where P is the ratio before and after applying the low pass filter, Q is the ratio before and after applying the baseline removal filter, and R is calculated by the computation of extreme point dispersion. In a study that evaluates SQI based on a template, the template for a normal-quality waveform is generated and compared with the input waveform to evaluate the quality. Orphanidou et al. (2014) proposed a method for evaluating waveform quality using HR, RR interval range, ratio of the maximum RR interval to the minimum RR interval, and result of adaptive template matching for ensemble average waveform of the whole pulsatile waveform. Li and Clifford (2012) normalized the length between the template and each pulse signal by dynamic time warping when matching the template. In their study, the template was created by ensemble-averaging pulsation waves within the first 30 s. Papini et al. (2018) used a template created by applying dynamic time warping barycenter averaging to PPG measured for an hour in template-based signal quality evaluation. Unlike existing methods, this template generation method does not require an alignment process for ensemble averaging. Thus, it offers more robust performance. Karlen et al. (2012) proposed a technique to evaluate signal quality in the range of 0–100 by calculating the cross correlation between successive beats and inputting a normalized cross-correlation coefficient to a nonlinear scaling function; this algorithm showed reasonable performance, but because it is based on exponential operation, it requires high computing power. In addition to the SQI evaluation method based on shape characteristics or templates of PPG or rule-based, studies on the SQI evaluation of PPG using machine learning have been actively conducted in recent years.

#### Machine Learning- and Deep Learning-Based Signal Quality Assessment

Liu et al. (2020b) evaluated PPG SQI using a five-layer fuzzy neural network. In their study, the quality of the signal was classified into three grades from the error of stroke volume measured with a commercial device. Stroke volume was calculated from PPG, and SQI evaluation performance was evaluated by inputting parameters extracted from PPG to the developed model. As a result, sensitivity of 0.81 and specificity 0.9 were shown for high-quality PPG, while sensitivity of 0.84 and specificity of 0.93 were shown for low-quality PPG. In another study by Liu et al. (2020a), PPG and derivative PPG were segmented for each beat and merged into a two-dimensional image to be used as input, and a machine learning model, a deep convolutional neural network (DCNN), VGG-19, or a residual DCNN (ResNet-50) was used to classify PPG segments into three grades of high, middle, and low. As a result, the study showed that the machine learning method using two-dimensional (2D) residual DCNN (RestNet-50) could more accurately evaluate signal quality than the method using general DCNN. Naeini et al. (2019) introduced a CNN-based method to evaluate the quality of PPG in an Internet-of-things-based health monitoring system. Their study performed binary classification of “reliable or unreliable” for PPG quality using an entire 60-s PPG signal as a CNN input, not extracted features, showing a precision of 0.89, and a recall of 0.83. Pradhan et al. (2019) conducted a study comparing the performance of five machine learning classifiers (k-nearest neighbor, multi-class support vector machine, naive Bayes, decision tree, and random forest) to evaluate the SQI of PPG using a wrist-wearable device. In their study, PPG quality was classified into five grades; it was found that the random forest SQI evaluation algorithm had the highest classification accuracy, with an accuracy of 74.5%. In a recent study, Guo et al. (2021) detected wearable PPG artifacts with a DICE score of 0.87–0.91 through a combination of active-contour-based loss and an adapted U-Net architecture; compared to the existing general research methods, this method shows superior performance. However, to sufficiently verify the performance of the deep-learning model, verification using more abundant data is required.

## Discussion

As seen in previous studies, most PPG pre-processing techniques rely on frequency domain filtering, which is effective in removing noise in a range that does not overlap with the core frequency of PPG. However, frequency domain filtering has limitations in handling non-stationary noise, making it possible to predict limitations that existing popular pre-processing technologies face when presuming increase in the use of PPG in future mobile environment. In the mobile environment, various types of non-stationary noise representing MAs can be introduced. This is expected to provide a completely different experience from measurement in an existing well-controlled environment. EMD or wavelet-based pre-processing technology can be a good alternative for dealing with frequency noise that does not overlap with the frequency component of PPG or non-stationary signals. However, it is also difficult to cope with severe distortion of the signal, such as saturation due to MAs and poor contact. Therefore, an innovative countermeasure against severe signal distortion and non-stationary dynamics is needed in the future by pursuing PPG pre-processing.

In relation to heartbeat, since PPG has a relatively simple waveform, the complexity of the pulsatile feature detection algorithm is relatively low for PPG signals compared to other physiological signals. The pulsatile feature point detection accuracy PPG may depend on the pre-processing algorithm with superior noise removal or waveform recovery performance rather than a pulsatile feature point detection algorithm. On the other hand, while the use of PPG intrapulse waveform feature continues to increase, there is no clearly verified detection algorithm. Nor is related research active. From this point of view, future PPG feature detection can be performed by focusing on an intrapulse feature detection algorithm related to differential pulse waves or dicrotic features rather than an algorithm that detects pulsatile features such as pulse onset and systolic peak.

Spatiotemporal features of PPG have already been analyzed in great detail for all inflection points of the waveform (Charlton et al., 2018; Yang et al., 2018; Mousavi et al., 2019). Therefore, rather than finding completely new morphological features from the PPG waveform, it may be more effective to find the clinical significance of existing features or discover a combination feature. However, as features become more sophisticated, more effort is required to detect a feature that might increase false detection rate. Therefore, when discovering and selecting features, it is important to keep in mind that the minimum number of features should always be used to obtain maximum effect. From this point of view, a machine learning-based analysis method that can estimate a specific result by inputting a raw signal without a special feature extraction process can have the potential for a new breakthrough for research on a PPG feature with increasing complexity.

For the representative noise of PPG, such as MAs, baseline wandering, and low perfusion, it has been reported that the baseline wandering noise of PPG can be effectively reduced with improved PPG detection performance through a relatively simple and easy-to-implement algorithm, such as a frequency filter or interpolation method. In addition, there have been attempts to decrease noise caused by low perfusion through hardware improvement, as well as software methods, such as adaptive filtering. However, a method that can completely remove the distortion of PPG waveform due to low perfusion remains unknown. Therefore, further research is needed to improve PPG distortion due to low perfusion. An approach from the viewpoint of noise removal technology and waveform reconstruction can be considered. MA as the most important topic of PPG signal processing can lead to the complete loss of PPG. Due to increased PPG measurement in a mobile environment, most in-depth research has been conducted for MA, compared to other noise factors. Despite various sophisticated algorithms that have been proposed for reducing MAs, a standard method for removing motion noise has yet to be introduced. Reviewing published studies on the removal of MAs revealed that an ICA method (Kim and Yoo, 2006), an acceleration sensor-based (Gibbs and Asada, 2005), and an adaptive filtering method using a Kalman filter (Seyedtabaii and Seyedtabaii, 2008) could be used. In the case of removing motion noise through simple frequency domain filtering using a high-pass filter, motion noise cannot be removed sensitively according to the intensity or type of motion. The ICA method and the adaptive filtering method can relatively improve the performance of motion noise removal. However, when the degree of motion noise is severe, they cannot be applied; for example, when the signal is saturated and completely lost because of motion. In such a case, a method of dividing the measured PPG into sections in which a signal exists, and a section in which the signal is lost, and analyzing selectively according to the classified section, has been proposed. In this method, the section in which motion noise can be removed is analyzed by applying motion noise removal technology, while the section in which motion noise could not be removed is excluded from analysis. Recently, research studies on SQI as an index for evaluating signal quality to distinguish between an analyzable section and an unanalyzable section have significantly increased. By evaluating the quality of a signal using SQI, false alarms in a patient monitoring device can be prevented, and the accuracy of clinical analysis can be improved by excluding error sections when interpreting signals. SQI is expected to be used in parallel with signal processing technology in the pre-processing stage. As mobile healthcare or wearable technology develops, its utilization will increase further. Machine learning technology is being applied in all areas of PPG signal processing, such as noise reduction, feature detection, and result analysis.

Machine learning in physiological analysis can omit complex and high error probability processing stages, such as feature detection, and derive results through end-to-end learning. This is expected to improve accuracy in analysis. For example, if a machine learning technique is applied, heart rate may be derived from the PPG signal itself, without other procedures, such as frequency domain transform, and peak detection or peak detection and feature detection can be excluded when deriving analytical results, such as SQI. In addition, since machine learning can be used to remove noise or generate new waveforms, its application to PPG processing is expected to increase in the future. Although machine learning is a promising method for analyzing PPG signals to be used in various applications, it is necessary to secure a highly relevant large data set and develop specialized models for each subdivided application. In particular, attempts to find meaningful information from PPG using various deep learning models are continuously increasing. Representative applications of PPG analysis using deep learning include heart rate estimation (Biswas et al., 2019; Reiss et al., 2019; Panwar et al., 2020; Chang et al., 2021; Mehrgardt et al., 2021), cuff-less blood pressure estimation (Panwar et al., 2020; El-Hajj and Kyriacou, 2021a,b; Schrumpf et al., 2021a,b; Tazarv and Levorato, 2021), and arterial fibrillation prediction (Poh et al., 2018; Kwon et al., 2019; Aschbacher et al., 2020; Cheng et al., 2020; Pereira et al., 2020). In addition, PPG-based deep learning models are being used for respiratory rate estimation (Ravichandran et al., 2019), sleep monitoring (Korkalainen et al., 2020), diabetes (Avram et al., 2019), vascular aging estimation (Dall’Olio et al., 2020), and peripheral arterial disease classification (Allen et al., 2021). In addition, to explain the causal relationship between input data and output results, an in-depth approach using technologies such as explainable AI, which has been recently studied, needs to be conducted. With respect to bio-signals, although explainable AI has been mainly applied to ECG (Sanjana et al., 2020; Ganeshkumar et al., 2021; Jo et al., 2021; Maweu et al., 2021; Raza et al., 2021; Taniguchi et al., 2021), it is difficult to find a clear application case for medical purposes in PPG. Although it is difficult to say that the application of explainable AI to PPG has been generalized yet, it seems clear that explainable AI will be introduced into PPG analysis given the tendency for the development of machine learning to be introduced into other fields. Machine learning is currently being continuously researched and developed. Finding and utilizing recent techniques and new methods, including explainable AI, will help in the analysis of PPG signals.

## Author Contributions

HSh contributed to the conception and design of the manuscript, and drafting, writing, and critical review of the final document. JP, HSe, and S-SK contributed to the literature search, data collection and analysis, drafting and writing, and figure design and drawing. All authors contributed to the article and approved the submitted version.

## Conflict of Interest

The authors declare that the research was conducted in the absence of any commercial or financial relationships that could be construed as a potential conflict of interest.

## Publisher’s Note

All claims expressed in this article are solely those of the authors and do not necessarily represent those of their affiliated organizations, or those of the publisher, the editors and the reviewers. Any product that may be evaluated in this article, or claim that may be made by its manufacturer, is not guaranteed or endorsed by the publisher.

## References

[B1] Adler J. N. Hughes L. A. Vtvilecchia R. Camargo A. C. Jr. (1998). Effect of skin pigmentation on pulse oximetry accuracy in the emergency department. *Acad. Emergency Med.* 5 965–970. 10.1111/j.1553-2712.1998.tb02772.x 9862586

[B2] Ahonen J. Jokela R. Uutela K. Huiku M. (2007). Surgical stress index reflects surgical stress in gynaecological laparoscopic day-case surgery. *Br. J. Anaesthesia* 98 456–461. 10.1093/bja/aem035 17350969

[B3] Aiba Y. Ohshiba S. Horiguchi S. I. Morioka I. Miyashita K. Kiyota I. (1999). Peripheral hemodynamics evaluated by acceleration plethysmography in workers exposed to lead. *Industrial Health* 37 3–8. 10.2486/indhealth.37.3 10052293

[B4] Alian A. A. Shelley K. H. (2014). Photoplethysmography. *Best Pract. Res. Clin. Anaesthesiol.* 28 395–406. 25480769 10.1016/j.bpa.2014.08.006

[B5] Allen J. (2007). Photoplethysmography and its application in clinical physiological measurement. *Physiol. Meas.* 28:R1. 10.1088/0967-3334/28/3/R01 17322588

[B6] Allen J. Liu H. Iqbal S. Zheng D. Stansby G. (2021). Deep learning-based photoplethysmography classification for peripheral arterial disease detection: a proof-of-concept study. *Physiol. Meas.* 42:054002. 10.1088/1361-6579/abf9f3 33878743

[B7] Allen J. Murray A. (1993). Development of a neural network screening aid for diagnosing lower limb peripheral vascular disease from photoelectric plethysmography pulse waveforms. *Physiol. Meas.* 14:13. 10.1088/0967-3334/14/1/003 8477229

[B8] Allen J. Murray A. (2000a). Similarity in bilateral photoplethysmographic peripheral pulse wave characteristics at the ears, thumbs and toes. *Physiol. Meas.* 21:369. 10.1088/0967-3334/21/3/303 10984205

[B9] Allen J. Murray A. (2000b). Variability of photoplethysmography peripheral pulse measurements at the ears, thumbs and toes. *IEEE Proc.-Sci. Meas. Technol.* 147 403–407.

[B10] Allen J. Murray A. (2002). Age-related changes in peripheral pulse timing characteristics at the ears, fingers and toes. *J. Hum. Hypertens.* 16 711–717. 10.1038/sj.jhh.1001478 12420195

[B11] Allen J. Murray A. (2004). “Effects of filtering on multisite photoplethysmography pulse waveform characteristics,” in *Proceeding of the Computers in Cardiology, 2004*, (IEEE), 485–488.

[B12] Alnaeb M. E. Alobaid N. Seifalian A. M. Mikhailidis D. P. Hamilton G. (2007). Optical techniques in the assessment of peripheral arterial disease. *Curr. Vasc. Pharmacol.* 5 53–59. 10.2174/157016107779317242 17266613

[B13] Alty S. R. Angarita-Jaimes N. Millasseau S. C. Chowienczyk P. J. (2007). Predicting arterial stiffness from the digital volume pulse waveform. *IEEE Trans. Biomed. Eng.* 54 2268–2275. 10.1109/tbme.2007.897805 18075043

[B14] Asada H. H. Shaltis P. Reisner A. Rhee S. Hutchinson R. C. (2003). Mobile monitoring with wearable photoplethysmographic biosensors. *IEEE Eng. Med. Biol. Magazine* 22 28–40. 10.1109/memb.2003.1213624 12845817

[B15] Aschbacher K. Yilmaz D. Kerem Y. Crawford S. Benaron D. Liu J. (2020). Atrial fibrillation detection from raw photoplethysmography waveforms: a deep learning application. *Heart Rhythm O2* 1 3–9. 10.1016/j.hroo.2020.02.002 34113853 PMC8183963

[B16] Avram R. Tison G. Kuhar P. Marcus G. Pletcher M. Olgin J. E. (2019). Predicting diabetes from photoplethysmography using deep learning. *J. Am. Coll. Cardiol.* 73 16–16. 10.3349/ymj.2022.63.S93 35040610 PMC8790582

[B17] Awad A. A. Haddadin A. S. Tantawy H. Badr T. M. Stout R. G. Silverman D. G. (2007). The relationship between the photoplethysmographic waveform and systemic vascular resistance. *J. Clin. Monit. Comput.* 21 365–372. 10.1007/s10877-007-9097-5 17940842

[B18] Ayata D. Yaslan Y. Kamasak M. E. (2018). Emotion based music recommendation system using wearable physiological sensors. *IEEE Tran. Consumer Electron.* 64 196–203. 10.1109/tce.2018.2844736

[B19] Baek H. J. Kim J. S. Kim Y. S. Lee H. B. Park K. S. (2007). “Second derivative of photoplethysmography for estimating vascular aging,” in *Proceeding of the 6th International Special Topic Conference on Information Technology Applications in Biomedicine*, (IEEE), 70–72. 10.1371/journal.pone.0135659

[B20] Baek H. J. Kim K. K. Kim J. S. Lee B. Park K. S. (2009). Enhancing the estimation of blood pressure using pulse arrival time and two confounding factors. *Physiol. Meas.* 31:145. 10.1088/0967-3334/31/2/002 20009186

[B21] Bagha S. Shaw L. (2011). A real time analysis of PPG signal for measurement of SpO2 and pulse rate. *Int. J. Comput. Appl.* 36 45–50.

[B22] Baker W. B. Parthasarathy A. B. Busch D. R. Mesquita R. C. Greenberg J. H. Yodh A. (2014). Modified Beer-Lambert law for blood flow. *Biomed. Optics Express* 5 4053–4075. 10.1364/BOE.5.004053 25426330 PMC4242038

[B23] Barnes R. W. Clayton J. M. Bone G. E. Slaymaker E. E. Reinertson J. (1977). Supraorbital photoplethysmography. Simple, accurate screening for carotid occlusive disease. *J. Surgical Res.* 22 319–327. 10.1016/0022-4804(77)90150-0 850395

[B24] Beer A. (1851). Versuch der absorptions-verhältnisse des cordierites für rothes licht zu bestimmen. *Ann. Physik Chem.* 84 37–52. 10.1002/andp.18511600904

[B25] Behar J. Roebuck A. Shahid M. Daly J. Hallack A. Palmius N. (2014). SleepAp: an automated obstructive sleep apnoea screening application for smartphones. *IEEE J. Biomed. Health Inform.* 19 325–331. 10.1109/JBHI.2014.2307913 25561453

[B26] Bentham M. Stansby G. Allen J. (2018). Innovative multi-site photoplethysmography analysis for quantifying pulse amplitude and timing variability characteristics in peripheral arterial disease. *Diseases* 6:81. 10.3390/diseases6030081 30227612 PMC6165367

[B27] Biswas D. Everson L. Liu M. Panwar M. Verhoef B.-E. Patki S. (2019). CorNET: deep learning framework for PPG-based heart rate estimation and biometric identification in ambulant environment. *IEEE Trans. Biomed. Circuits Syst.* 13 282–291. 10.1109/TBCAS.2019.2892297 30629514

[B28] Bohusch T. Miller T. Kreamer W. Eisenhardt J. R. Foehl H. C. (1994). Correlation of photoplethysmography, doppler velocimetry, digital blood pressure and skin temperature in the hand under conditions of warming. *Cardiopulmonary Phys. Ther. J.* 5 15–18.

[B29] Bortolotto L. A. Blacher J. Kondo T. Takazawa K. Safar M. E. (2000). Assessment of vascular aging and atherosclerosis in hypertensive subjects: second derivative of photoplethysmogram versus pulse wave velocity. *Am. J. Hypertens.* 13 165–171. 10.1016/s0895-7061(99)00192-2 10701816

[B30] Brillante D. G. O’sullivan A. J. Howes L. G. (2008). Arterial stiffness indices in healthy volunteers using non-invasive digital photoplethysmography. *Blood Pressure* 17 116–123. 10.1080/08037050802059225 18568701

[B31] Canac N. Ranjbaran M. O’brien M. J. Asgari S. Scalzo F. Thorpe S. G. (2019). Algorithm for reliable detection of pulse onsets in cerebral blood flow velocity signals. *Front. Neurol.* 10:1072. 10.3389/fneur.2019.01072 31681147 PMC6798080

[B32] Cannesson M. Besnard C. Durand P. G. Bohé J. Jacques D. (2005). Relation between respiratory variations in pulse oximetry plethysmographic waveform amplitude and arterial pulse pressure in ventilated patients. *Crit. Care* 9:R562. 10.1186/cc3799 16277719 PMC1297625

[B33] Cannesson M. Desebbe O. Rosamel P. Delannoy B. Robin J. Bastien O. (2008b). Pleth variability index to monitor the respiratory variations in the pulse oximeter plethysmographic waveform amplitude and predict fluid responsiveness in the operating theatre. *Br. J. Anaesthesia* 101 200–206. 10.1093/bja/aen133 18522935

[B34] Cannesson M. Delannoy B. Morand A. Rosamel P. Attof Y. Bastien O. (2008a). Does the pleth variability index indicate the respiratory-induced variation in the plethysmogram and arterial pressure waveforms? *Anesthesia Analgesia* 106 1189–1194. 10.1213/ane.0b013e318167ab1f 18349191

[B35] Challoner A. (1979). Photoelectric plethysmography for estimating cutaneous blood flow. *Non-Invasive Physiol. Meas.* 1 125–151.

[B36] Chang X. Li G. Xing G. Zhu K. Tu L. (2021). DeepHeart: a deep learning approach for accurate heart rate estimation from PPG signals. *ACM Trans. Sensor Netw. (TOSN)* 17 1–18.

[B37] Charlton P. H. Celka P. Farukh B. Chowienczyk P. Alastruey J. (2018). Assessing mental stress from the photoplethysmogram: a numerical study. *Physiol. Meas.* 39:054001. 10.1088/1361-6579/aabe6a 29658894 PMC5964362

[B38] Chen W. Kobayashi T. Ichikawa S. Takeuchi Y. Togawa T. (2000). Continuous estimation of systolic blood pressure using the pulse arrival time and intermittent calibration. *Med. Biol. Eng. Comput.* 38 569–574. 10.1007/BF02345755 11094816

[B39] Cheng P. Chen Z. Li Q. Gong Q. Zhu J. Liang Y. (2020). Atrial fibrillation identification with PPG signals using a combination of time-frequency analysis and deep learning. *IEEE Access* 8 172692–172706.

[B40] Choi B. Park C. Lee Y. Shin H. Lee S. Jeong S. (2018). Development of a new analgesic index using nasal photoplethysmography. *Anaesthesia* 73 1123–1130. 10.1111/anae.14327 29790159

[B41] Choi J. Ahmed B. Gutierrez-Osuna R. (2011). Development and evaluation of an ambulatory stress monitor based on wearable sensors. *IEEE Trans. Inform. Technol. Biomed.* 16 279–286. 10.1109/TITB.2011.2169804 21965215

[B42] Chu C. L. Huang Y. Y. Chen Y. H. Lai L. P. Yeh H. M. (2018). An observational study: the utility of perfusion index as a discharge criterion for pain assessment in the postanesthesia care unit. *PLoS One* 13:e0197630. 10.1371/journal.pone.0197630 29768487 PMC5955537

[B43] Chua C. Heneghan C. (2006). “Continuous blood pressure monitoring using ECG and finger photoplethysmogram,” in *Proceeding of the International Conference of the IEEE Engineering in Medicine and Biology Society*, (IEEE), 5117–5120. 10.1109/IEMBS.2006.259612 17946678

[B44] Dahlgren G. Veintemilla F. Settergren G. Liska J. (1991). Left ventricular end-systolic pressure estimated from measurements in a peripheral artery. *J. Cardiothor. Vasc. Anesthesia* 5 551–553. 10.1016/1053-0770(91)90004-d 1768817

[B45] Dall’Olio L. Curti N. Remondini D. Harb Y. S. Asselbergs F. W. Castellani G. (2020). Prediction of vascular aging based on smartphone acquired PPG signals. *Sci. Rep.* 10:19756. 10.1038/s41598-020-76816-6 33184391 PMC7661535

[B46] De Trafford J. Lafferty K. (1984). What does photoplethysmography measure? *Med. Biol. Eng. Comput.* 22 479–480. 10.1007/BF02447713 6482540

[B47] Dillon J. B. Hertzman A. B. (1941). The form of the volume pulse in the finger pad in health, arteriosclerosis, and hypertension. *Am. Heart J.* 21 172–190. 10.1016/s0002-8703(41)90966-3

[B48] Ding X. Yan B. P. Zhang Y.-T. Liu J. Zhao N. Tsang H. K. (2017). Pulse transit time based continuous cuffless blood pressure estimation: a new extension and a comprehensive evaluation. *Sci. Rep.* 7:11554. 10.1038/s41598-017-11507-3 28912525 PMC5599606

[B49] Ding X.-R. Zhang Y.-T. (2015). “Photoplethysmogram intensity ratio: a potential indicator for improving the accuracy of PTT-based cuffless blood pressure estimation,” in *Proceeding of the 37th Annual International Conference of the IEEE Engineering in Medicine and Biology Society (EMBC)*, (IEEE), 398–401. 10.1109/EMBC.2015.7318383 26736283

[B50] Dorlas J. Nijboer J. (1985). Photo-electric plethysmography as a monitoring device in anaesthesia: application and interpretation. *Br. J. Anaesthesia* 57 524–530. 10.1093/bja/57.5.524 3994887

[B51] Dresher R. P. Mendelson Y. (2006b). “Reflectance forehead pulse oximetry: effects of contact pressure during walking,” in *Proceeding of the International Conference of the IEEE Engineering in Medicine and Biology Society*, (IEEE), 3529–3532. 10.1109/IEMBS.2006.260136 17946185

[B52] Dresher R. P. Mendelson Y. (2006a). “A new reflectance pulse oximeter housing to reduce contact pressure effects,” in *Proceedings of the IEEE 32nd Annual Northeast Bioengineering Conference*, (IEEE), 49–50.

[B53] Eerikäinen L. M. Bonomi A. G. Schipper F. Dekker L. R. De Morree H. M. Vullings R. (2019). Detecting atrial fibrillation and atrial flutter in daily life using photoplethysmography data. *IEEE J. Biomed. Health Inform.* 24 1610–1618. 10.1109/JBHI.2019.2950574 31689222

[B54] El Hajj C. Kyriacou P. A. (2020). “Cuffless and continuous blood pressure estimation from ppg signals using recurrent neural networks,” in *Proceeding of the 42nd Annual International Conference of the IEEE Engineering in Medicine & Biology Society (EMBC)*, (IEEE), 4269–4272. 10.1109/EMBC44109.2020.9175699 33018939

[B55] Elgendi M. (2016). Optimal signal quality index for photoplethysmogram signals. *Bioengineering* 3:21. 10.3390/bioengineering3040021 28952584 PMC5597264

[B56] El-Hajj C. Kyriacou P.A. (2021a). Cuffless blood pressure estimation from PPG signals and its derivatives using deep learning models. *Biomed. Signal Process. Control* 70:102984.

[B57] El-Hajj C. Kyriacou P. A. (2021b). Deep learning models for cuffless blood pressure monitoring from PPG signals using attention mechanism. *Biomed. Signal Process. Control* 65:102301.

[B58] Fallow B. A. Tarumi T. Tanaka H. (2013). Influence of skin type and wavelength on light wave reflectance. *J. Clin. Monit. Comput.* 27 313–317. 10.1007/s10877-013-9436-7 23397431

[B59] Ferro B. R. Aguilera A. R. De La Vara Prieto R. F. (2015). Automated detection of the onset and systolic peak in the pulse wave using Hilbert transform. *Biomed. Signal Process. Control* 20 78–84. 10.1016/j.bspc.2015.04.009

[B60] Fischer C. Glos M. Penzel T. Fietze I. (2017). Extended algorithm for real-time pulse waveform segmentation and artifact detection in photoplethysmograms. *Somnologie* 21 110–120. 10.1109/JBHI.2016.2518202 26780821

[B61] Foo J. Y. A. (2007). Development of a temperature-controlled miniature enclosure for monitoring poor perfusion photoplethysmographic signals. *Physiol. Meas.* 28:N67. 10.1088/0967-3334/28/9/N01 17827645

[B62] Foo J. Y. A. Lim C. S. Wang P. (2006). Evaluation of blood pressure changes using vascular transit time. *Physiol. Meas.* 27:685. 10.1088/0967-3334/27/8/003 16772667

[B63] Foo J. Y. A. Wilson S. J. (2006). A computational system to optimise noise rejection in photoplethysmography signals during motion or poor perfusion states. *Med. Biol. Eng. Comput.* 44 140–145. 10.1007/s11517-005-0008-y 16929932

[B64] Freckmann G. Pleus S. Haug C. Bitton G. Nagar R. (2012). Increasing local blood flow by warming the application site: beneficial effects on postprandial glycemic excursions. *J. Diabetes Sci. Technol.* 6 780–785. 10.1177/193229681200600407 22920802 PMC3440147

[B65] Fu T.-H. Liu S.-H. Tang K.-T. (2008). Heart rate extraction from photoplethysmogram waveform using wavelet multi-resolution analysis. *J. Med. Biol. Eng.* 28 229–232.

[B66] Fung P. Dumont G. Ries C. Mott C. Ansermino M. (2004). “Continuous noninvasive blood pressure measurement by pulse transit time,” in *Proceeding of the 26th Annual International Conference of the IEEE Engineering in Medicine and Biology Society*, (IEEE), 738–741. 10.1109/IEMBS.2004.1403264 17271783

[B67] Ganeshkumar M. Ravi V. Sowmya V. Gopalakrishnan E. Soman K. (2021). “Explainable deep learning-based approach for multilabel classification of electrocardiogram,” in *Proceeding of the IEEE Transactions on Engineering Management*, (IEEE).

[B68] Gibbs P. Asada H. H. (2005). “Reducing motion artifact in wearable bio-sensors using MEMS accelerometers for active noise cancellation,” in *Proceedings of the 2005, American Control Conference, 2005*, (IEEE), 1581–1586.

[B69] Gil E. Orini M. Bailón R. Vergara J. M. Mainardi L. Laguna P. (2010). Photoplethysmography pulse rate variability as a surrogate measurement of heart rate variability during non-stationary conditions. *Physiol. Meas.* 31 1271–1290. 10.1088/0967-3334/31/9/015 20702919

[B70] Goshvarpour A. Goshvarpour A. (2018). Poincaré’s section analysis for PPG-based automatic emotion recognition. *Chaos Solitons Fractals* 114 400–407. 10.1016/j.chaos.2018.07.035

[B71] Goshvarpour A. Goshvarpour A. (2020). Evaluation of novel entropy-based complex wavelet sub-bands measures of PPG in an emotion recognition system. *J. Med. Biol. Eng.* 40 451–461.

[B72] Guo Y. Wang H. Zhang H. Liu T. Liang Z. Xia Y. (2019). Mobile photoplethysmographic technology to detect atrial fibrillation. *J. Am. College Cardiol.* 74 2365–2375. 10.1016/j.jacc.2019.08.019 31487545

[B73] Guo Z. Ding C. Hu X. Rudin C. (2021). A supervised machine learning semantic segmentation approach for detecting artifacts in plethysmography signals from wearables. *Physiol. Meas.* 42:125003. 10.1088/1361-6579/ac3b3d 34794126

[B74] Han H. Kim M.-J. Kim J. (2007). “Development of real-time motion artifact reduction algorithm for a wearable photoplethysmography,” in *Proceeding of the 29th Annual International Conference of the IEEE Engineering in Medicine and Biology Society*, (IEEE), 1538–1541. 10.1109/IEMBS.2007.4352596 18002262

[B75] Hartmut Gehring M. Me H. M. Schmucker P. (2002). The effects of motion artifact and low perfusion on the performance of a new generation of pulse oximeters in volunteers undergoing hypoxemia. *Respiratory Care* 47 48–60. 10.1097/00132586-199802000-00051 11749687

[B76] Hasanin A. Mohamed S. A. R. El-Adawy A. (2017). Evaluation of perfusion index as a tool for pain assessment in critically ill patients. *J. Clin. Monit. Comput.* 31 961–965. 10.1007/s10877-016-9936-3 27665572

[B77] He J.-L. Lecarpentier Y. Zamani K. Coirault C. Daccache G. Chemla D. (1995). Relation between aortic dicrotic notch pressure and mean aortic pressure in adults. *Am. J. Cardiol.* 76 301–306. 10.1016/s0002-9149(99)80086-1 7618629

[B78] He X. Goubran R. A. Liu X. P. (2014). Secondary peak detection of PPG signal for continuous cuffless arterial blood pressure measurement. *IEEE Trans. Instrument. Meas.* 63 1431–1439. 10.1109/tim.2014.2299524

[B79] Hertzman A. B. (1937). Observations on the finger volume pulse recorded photoelectrically. *Am. J. Physiol.* 119 334–335.

[B80] Hertzman A. B. (1938). The blood supply of various skin areas as estimated by the photoelectric plethysmograph. *Am. J. Physiol.-Legacy Content* 124 328–340.

[B81] Hertzman A. B. Dillon J. B. (1940). Applications of photoelectric plethysmography in peripheral vascular disease. *Am. Heart J.* 20 750–761. 10.1016/s0002-8703(40)90534-8

[B82] Hertzman A. B. Roth L. W. (1942). The absence of vasoconstrictor reflexes in the forehead circulation. Effects of cold. *Am. J. Physiol.-Legacy Content* 136 692–697.

[B83] Hilmisson H. Berman S. Magnusdottir S. (2020). Sleep apnea diagnosis in children using software-generated apnea-hypopnea index (AHI) derived from data recorded with a single photoplethysmogram sensor (PPG). *Sleep Breath.* 24 1739–1749. 10.1007/s11325-020-02049-6 32222900

[B84] Hummler H. D. Engelmann A. Pohlandt F. Högel J. Franz A. R. (2006). Decreased accuracy of pulse oximetry measurements during low perfusion caused by sepsis: is the perfusion index of any value? *Intensive Care Med.* 32 1428–1431. 10.1007/s00134-006-0254-y 16810522

[B85] Imanaga I. Hara H. Koyanagi S. Tanaka K. (1998). Correlation between wave components of the second derivative of plethysmogram and arterial distensibility. *Japanese Heart J.* 39 775–784. 10.1536/ihj.39.775 10089939

[B86] Jang D.-G. Farooq U. Park S.-H. Hahn M. (2014). A robust method for pulse peak determination in a digital volume pulse waveform with a wandering baseline. *IEEE Trans. Biomed. Circuits Syst.* 8 729–737. 10.1109/TBCAS.2013.2295102 25388880

[B87] Jo Y.-Y. Cho Y. Lee S. Y. Kwon J.-M. Kim K.-H. Jeon K.-H. (2021). Explainable artificial intelligence to detect atrial fibrillation using electrocardiogram. *Int. J. Cardiol.* 328 104–110. 10.1016/j.ijcard.2020.11.053 33271204

[B88] Jones D. P. (1987). Medical electro-optics: measurements in the human microcirculation. *Phys. Technol.* 18:79. 10.1515/jbcpp.1992.3.3.193 1298339

[B89] Joseph G. Joseph A. Titus G. Thomas R. M. Jose D. (2014). “Photoplethysmogram (PPG) signal analysis and wavelet de-noising,” in *Proceeding of the Annual International Conference on Emerging Research Areas: Magnetics, Machines and Drives (AICERA/iCMMD)*, (IEEE), 1–5.

[B90] Jubadi W. M. Sahak S. F. A. M. (2009). “Heartbeat monitoring alert via SMS,” in *Proceeding of the IEEE Symposium on Industrial Electronics & Applications*, (IEEE), 1–5. 10.9734/bjast/2016/28453

[B91] Kallio H. Lindberg L. Majander A. Uutela K. Niskanen M. Paloheimo M. (2008). Measurement of surgical stress in anaesthetized children. *Br. J. Anaesthesia* 101 383–389. 10.1093/bja/aen204 18628266

[B92] Kamal A. Harness J. Irving G. Mearns A. (1989). Skin photoplethysmography—a review. *Comput. Methods Programs Biomed.* 28 257–269. 10.1016/0169-2607(89)90159-4 2649304

[B93] Karlen W. Kobayashi K. Ansermino J. M. Dumont G. (2012). Photoplethysmogram signal quality estimation using repeated Gaussian filters and cross-correlation. *Physiol. Meas.* 33:1617. 10.1088/0967-3334/33/10/1617 22986287

[B94] Karlen W. Raman S. Ansermino J. M. Dumont G. A. (2013). Multiparameter respiratory rate estimation from the photoplethysmogram. *IEEE Trans. Biomed. Eng.* 60 1946–1953. 10.1109/TBME.2013.2246160 23399950

[B95] Kavsaoğlu A. R. Polat K. Bozkurt M. R. (2016). An innovative peak detection algorithm for photoplethysmography signals: an adaptive segmentation method. *Turkish J. Electrical Eng. Comput. Sci.* 24 1782–1796. 10.3906/elk-1310-177 31411186

[B96] Khan M. Pretty C. G. Amies A. C. Elliott R. Chiew Y. S. Shaw G. M. (2016). Analysing the effects of cold, normal, and warm digits on transmittance pulse oximetry. *Biomed. Signal Process. Control* 26 34–41. 10.1016/j.bspc.2015.12.006

[B97] Kim B. S. Yoo S. K. (2006). Motion artifact reduction in photoplethysmography using independent component analysis. *IEEE Trans. Biomed. Eng.* 53 566–568. 10.1109/TBME.2005.869784 16532785

[B98] Kim J. Kim J. Ko H. (2016). Low-power photoplethysmogram acquisition integrated circuit with robust light interference compensation. *Sensors* 16:46. 10.3390/s16010046 26729122 PMC4732079

[B99] Kim J. Lee J.-W. Shin H. (2019). Pre-processing of photoplethysmographic waveform for amplitude regularization. *J. Electrical Eng. Technol.* 14 1741–1748.

[B100] Kim J. Lee T. Kim J. Ko H. (2015). “Ambient light cancellation in photoplethysmogram application using alternating sampling and charge redistribution technique,” in *Proceeding of the 37th Annual International Conference of the IEEE Engineering in Medicine and Biology Society (EMBC)*, (IEEE), 6441–6444. 10.1109/EMBC.2015.7319867 26737767

[B101] Korhonen I. Yli-Hankala A. (2009). Photoplethysmography and nociception. *Acta Anaesthesiol. Scandinavica* 53 975–985. 10.1111/j.1399-6576.2009.02026.x 19572939

[B102] Korkalainen H. Aakko J. Duce B. Kainulainen S. Leino A. Nikkonen S. (2020). Deep learning enables sleep staging from photoplethysmogram for patients with suspected sleep apnea. *Sleep* 43:zsaa098. 10.1093/sleep/zsaa098 32436942 PMC7658638

[B103] Krishnan R. Natarajan B. Warren S. (2008). “Motion artifact reduction in photopleythysmography using magnitude-based frequency domain independent component analysis,” in *Proceedings of the 17th International Conference on Computer Communications and Networks*, (IEEE), 1–5.

[B104] Krishnaswamy A. Baranoski G. V. (2004). A biophysically-based spectral model of light interaction with human skin. *Comput. Graphics Forum* 23 331–340. 10.1111/j.1467-8659.2004.00764.x

[B105] Kwon S. Hong J. Choi E.-K. Lee E. Hostallero D. E. Kang W. J. (2019). Deep learning approaches to detect atrial fibrillation using photoplethysmographic signals: algorithms development study. *JMIR mHealth uHealth* 7:e12770. 10.2196/12770 31199302 PMC6592499

[B106] Kyriacou P. Powell S. Langford R. Jones D. (2002). Investigation of oesophageal photoplethysmographic signals and blood oxygen saturation measurements in cardiothoracic surgery patients. *Physiol. Meas.* 23:533. 10.1088/0967-3334/23/3/305 12214761

[B107] Lakshmanan S. Chatterjee D. Muniyandi M. (2018). “Noninvasive assistive method to diagnose arterial disease-takayasu’s arteritis,” in *Computational Vision and Bio Inspired Computing*, eds Hemanth D. J. Smys S. (Berlin: Springer), 384–398. 10.1007/978-3-319-71767-8_32

[B108] Landsverk S. A. Hoiseth L. O. Kvandal P. Hisdal J. Skare O. Kirkeboen K. A. (2008). Poor agreement between respiratory variations in pulse oximetry photoplethysmographic waveform amplitude and pulse pressure in intensive care unit patients. *Anesthesiol.: J. Am. Soc. Anesthesiol.* 109 849–855. 10.1097/ALN.0b013e3181895f9f 18946297

[B109] Lazazzera R. Deviaene M. Varon C. Buyse B. Testelmans D. Laguna P. (2020). Detection and classification of sleep apnea and hypopnea using PPG and SpO $ _2 $ signals. *IEEE Trans. Biomed. Eng.* 68 1496–1506. 10.1109/tbme.2020.3028041 32997622

[B110] Lee C. Shin H. S. Lee M. (2011a). Relations between ac-dc components and optical path length in photoplethysmography. *J. Biomed. Optics* 16:077012. 10.1117/1.3600769 21806292

[B111] Lee C. Shin H. S. Park J. Lee M. (2011b). The optimal attachment position for a fingertip photoplethysmographic sensor with low DC. *IEEE Sensors J.* 12 1253–1254.

[B112] Lee Q. Y. Chan G. S. Redmond S. J. Middleton P. M. Steel E. Malouf P. (2011c). Multivariate classification of systemic vascular resistance using photoplethysmography. *Physiol. Meas.* 32:1117. 10.1088/0967-3334/32/8/008 21693795

[B113] Lee J. Kim M. Park H.-K. Kim I. Y. (2020). Motion artifact reduction in wearable photoplethysmography based on multi-channel sensors with multiple wavelengths. *Sensors* 20:1493. 10.3390/s20051493 32182772 PMC7085621

[B114] Lee M. S. Lee Y. K. Pae D. S. Lim M. T. Kim D. W. Kang T. K. (2019). Fast emotion recognition based on single pulse PPG signal with convolutional neural network. *Appl. Sci.* 9:3355. 10.3390/app9163355

[B115] Lee Q. Y. Redmond S. J. Chan G. S. Middleton P. M. Steel E. Malouf P. (2013). Estimation of cardiac output and systemic vascular resistance using a multivariate regression model with features selected from the finger photoplethysmogram and routine cardiovascular measurements. *Biomed. Eng. Online* 12:19. 10.1186/1475-925X-12-19 23452705 PMC3649882

[B116] Li Q. Clifford G. D. (2012). Dynamic time warping and machine learning for signal quality assessment of pulsatile signals. *Physiol. Meas.* 33:1491. 10.1088/0967-3334/33/9/1491 22902950

[B117] Li S. Liu L. Wu J. Tang B. Li D. (2018). Comparison and noise suppression of the transmitted and reflected photoplethysmography signals. *BioMed. Res. Int.* 2018:4523593. 10.1155/2018/4523593 30356404 PMC6178150

[B118] Li X. Chen J. Zhao G. Pietikainen M. (2014). “Remote heart rate measurement from face videos under realistic situations,” in *Proceedings of the IEEE Conference on Computer Vision and Pattern Recognition*, (IEEE), 4264–4271.

[B119] Lima A. Bakker J. (2006). “Noninvasive monitoring of peripheral perfusion,” in *Applied Physiology in Intensive Care Medicine*, eds Pinsky M. R. Brochard L. Mancebo J. (Berlin: Springer), 131–141. 10.1007/3-540-37363-2_26

[B120] Lin Y.-H. Lin C.-F. You H.-Z. (2011). “A driver’s physiological monitoring system based on a wearable PPG sensor and a smartphone,” in *Proceeding of the International Conference on Security-Enriched Urban Computing and Smart Grid*, (Springer), 326–335. 10.1007/978-3-642-23948-9_36

[B121] Lindberg L.-G. Oberg P. A. (1993). Optical properties of blood in motion. *Optical Eng.* 32 253–258. 10.1117/12.60688

[B122] Linder S. P. Wendelken S. M. Wei E. Mcgrath S. P. (2006). Using the morphology of photoplethysmogram peaks to detect changes in posture. *J. Clin. Monitor. Comput.* 20 151–158. 10.1007/s10877-006-9015-2 16688391

[B123] Liu J. Li Y. Ding X.-R. Dai W.-X. Zhang Y.-T. (2015). “Effects of cuff inflation and deflation on pulse transit time measured from ECG and multi-wavelength PPG,” in *Proceeding of the 37th Annual International Conference of the IEEE Engineering in Medicine and Biology Society (EMBC)*, (IEEE), 5973–5976. 10.1109/EMBC.2015.7319752 26737652

[B124] Liu J. Yan B. P. Zhang Y.-T. Ding X.-R. Su P. Zhao N. (2018). Multi-wavelength photoplethysmography enabling continuous blood pressure measurement with compact wearable electronics. *IEEE Trans. Biomed. Eng.* 66 1514–1525. 10.1109/TBME.2018.2874957 30307851

[B125] Liu J. Yan B. P.-Y. Dai W.-X. Ding X.-R. Zhang Y.-T. Zhao N. (2016a). Multi-wavelength photoplethysmography method for skin arterial pulse extraction. *Biomed. Optics Express* 7 4313–4326. 10.1364/BOE.7.004313 27867733 PMC5102532

[B126] Liu J. Zhang Y.-T. Ding X.-R. Dai W.-X. Zhao N. (2016b). “A preliminary study on multi-wavelength PPG based pulse transit time detection for cuffless blood pressure measurement,” in *Proceeding of the 38th Annual International Conference of the IEEE Engineering in Medicine and Biology Society (EMBC)*, (IEEE), 615–618. 10.1109/EMBC.2016.7590777 28324936

[B127] Liu S.-H. Li R.-X. Wang J.-J. Chen W. Su C.-H. (2020a). Classification of photoplethysmographic signal quality with deep convolution neural networks for accurate measurement of cardiac stroke volume. *Appl. Sci.* 10:4612.

[B128] Liu S.-H. Wang J.-J. Chen W. Pan K.-L. Su C.-H. (2020b). Classification of photoplethysmographic signal quality with fuzzy neural network for improvement of stroke volume measurement. *Appl. Sci.* 10:1476. 10.3390/app10041476

[B129] Lu S. Zhao H. Ju K. Shin K. Lee M. Shelley K. (2008). Can photoplethysmography variability serve as an alternative approach to obtain heart rate variability information? *J. Clin. Monit. Comput.* 22 23–29. 10.1007/s10877-007-9103-y 17987395

[B130] Ma H. T. (2014). A blood pressure monitoring method for stroke management. *BioMed Res. Int.* 2014:571623. 10.1155/2014/571623 25197651 PMC4150505

[B131] Madhav K. V. Ram M. R. Krishna E. H. Komalla N. R. Reddy K. A. (2011). “Estimation of respiration rate from ECG, BP and PPG signals using empirical mode decomposition,” in *Proceeding of the IEEE International Instrumentation and Measurement Technology Conference*, (IEEE), 1–4.

[B132] Mase M. Mattei W. Cucino R. Faes L. Nollo G. (2011). Feasibility of cuff-free measurement of systolic and diastolic arterial blood pressure. *J. Electrocardiol.* 44 201–207. 10.1016/j.jelectrocard.2010.11.019 21353067

[B133] Matsumura K. Toda S. Kato Y. (2020). RGB and near-infrared light reflectance/transmittance photoplethysmography for measuring heart rate during motion. *IEEE Access* 8 80233–80242. 10.1109/access.2020.2990438

[B134] Maweu B. M. Dakshit S. Shamsuddin R. Prabhakaran B. (2021). CEFEs: a CNN explainable framework for ECG signals. *Artif. Intell. Med.* 115:102059. 10.1016/j.artmed.2021.102059 34001319

[B135] McCombie D. Asada H. Reisner A. (2005). “Identification of vascular dynamics and estimation of the cardiac output waveform from wearable PPG sensors,” in *Proceedings of the 2005 IEEE Engineering in Medicine and Biology 27th Annual Conference* (Piscataway, NJ: IEEE), 3490–3493. 10.1109/IEMBS.2005.1617231 17280976

[B136] McKay N. D. Griffiths B. Di Maria C. Hedley S. Murray A. Allen J. (2014). Novel photoplethysmography cardiovascular assessments in patients with Raynaud’s phenomenon and systemic sclerosis: a pilot study. *Rheumatology* 53 1855–1863. 10.1093/rheumatology/keu196 24850874

[B137] Mehrgardt P. Khushi M. Poon S. Withana A. (2021). Deep learning fused wearable pressure and PPG data for accurate heart rate monitoring. *IEEE Sens. J.* 21 27106–27115.

[B138] Mejía-Mejía E. Budidha K. Abay T. Y. May J. M. Kyriacou P. A. (2020). Heart rate variability (HRV) and pulse rate variability (PRV) for the assessment of autonomic responses. *Front. Physiol.* 11:779. 10.3389/fphys.2020.00779 32792970 PMC7390908

[B139] Millasseau S. C. Kelly R. Ritter J. Chowienczyk P. (2002). Determination of age-related increases in large artery stiffness by digital pulse contour analysis. *Clin. Sci.* 103 371–377. 10.1042/cs1030371 12241535

[B140] Millasseau S. C. Kelly R. P. Ritter J. M. Chowienczyk P. J. (2003). The vascular impact of aging andvasoactive drugs: comparison of two digital volume pulse measurements. *Am. J. Hypertens.* 16 467–472. 10.1016/s0895-7061(03)00569-7 12799095

[B141] Millasseau S. C. Ritter J. M. Takazawa K. Chowienczyk P. J. (2006). Contour analysis of the photoplethysmographic pulse measured at the finger. *J. Hypertens.* 24 1449–1456. 10.1097/01.hjh.0000239277.05068.87 16877944

[B142] Monnet X. Lamia B. Teboul J.-L. (2005). Pulse oximeter as a sensor of fluid responsiveness: do we have our finger on the best solution? *Crit. Care* 9:429. 10.1186/cc3876 16277729 PMC1297637

[B143] Mousavi S. S. Firouzmand M. Charmi M. Hemmati M. Moghadam M. Ghorbani Y. (2019). Blood pressure estimation from appropriate and inappropriate PPG signals using A whole-based method. *Biomed. Signal Process. Control* 47 196–206. 10.1016/j.bspc.2018.08.022

[B144] Mowafi H. A. Arab S. A. Ismail S. A. Al-Ghamdi A. A. Al-Metwalli R. R. (2008). Plethysmographic pulse wave amplitude is an effective indicator for intravascular injection of epinephrine-containing epidural test dose in sevoflurane-anesthetized pediatric patients. *Anesthesia Analgesia* 107 1536–1541. 10.1213/ane.0b013e3181844d08 18931211

[B145] Mowafi H. A. Ismail S. A. Shafi M. A. Al-Ghamdi A. A. (2009). The efficacy of perfusion index as an indicator for intravascular injection of epinephrine-containing epidural test dose in propofol-anesthetized adults. *Anesthesia Analgesia* 108 549–553. 10.1213/ane.0b013e31818fc35b 19151286

[B146] Muehlsteff J. Aubert X. Schuett M. (2006). “Cuffless estimation of systolic blood pressure for short effort bicycle tests: the prominent role of the pre-ejection period,” in *International Conference of the IEEE Engineering in Medicine and Biology Society*, (IEEE), 5088–5092. 10.1109/IEMBS.2006.260275 17946673

[B147] Murray W. B. Foster P. A. (1996). The peripheral pulse wave: information overlooked. *J. Clin. Monit.* 12 365–377. 10.1007/BF02077634 8934343

[B148] Nabeel P. Jayaraj J. Mohanasankar S. (2017). Single-source PPG-based local pulse wave velocity measurement: a potential cuffless blood pressure estimation technique. *Physiol. Meas.* 38:2122. 10.1088/1361-6579/aa9550 29058686

[B149] Naeini E. K. Azimi I. Rahmani A. M. Liljeberg P. Dutt N. (2019). A Real-time PPG quality assessment approach for healthcare internet-of-things. *Procedia Comput. Sci.* 151 551–558. 10.1016/j.procs.2019.04.074

[B150] Nilsson L. Johansson A. Kalman S. (2003a). Macrocirculation is not the sole determinant of respiratory induced variations in the reflection mode photoplethysmographic signal. *Physiol. Meas.* 24:925. 10.1088/0967-3334/24/4/009 14658783

[B151] Nilsson L. Johansson A. Kalman S. (2003b). Respiratory variations in the reflection mode photoplethysmographic signal. Relationships to peripheral venous pressure. *Med. Biol. Eng. Comput.* 41 249–254. 10.1007/BF02348428 12803288

[B152] Nitzan M. Babchenko A. Faib I. Davidson E. Adler D. (2000). “Assessment of changes in arterial compliance by photoplethysmography,” in *Proceeding of the 21st IEEE Convention of the Electrical and Electronic Engineers in Israel. Proceedings (Cat. No. 00EX377)*, (IEEE), 351–354.

[B153] Nitzan M. Faib I. Friedman H. (2006). Respiration-induced changes in tissue blood volume distal to occluded artery, measured by photoplethysmography. *J. Biomed. Optics* 11:040506. 10.1117/1.2236285 16965128

[B154] Nitzan M. Romem A. Koppel R. (2014). Pulse oximetry: fundamentals and technology update. *Med. Devices (Auckland, NZ)* 7:231. 10.2147/MDER.S47319 25031547 PMC4099100

[B155] Orjuela-Cañón A. D. Delisle-Rodríguez D. López-Delis A. De La Vara-Prieto R. F. Cuadra-Sanz M. B. (2013). “Onset and peak pattern recognition on photoplethysmographic signals using neural networks,” in *Proceeding of the Iberoamerican Congress on Pattern Recognition*, (Springer), 543–550.

[B156] Orphanidou C. Bonnici T. Charlton P. Clifton D. Vallance D. Tarassenko L. (2014). Signal-quality indices for the electrocardiogram and photoplethysmogram: derivation and applications to wireless monitoring. *IEEE J. Biomed. Health Inform.* 19 832–838. 10.1109/JBHI.2014.2338351 25069129

[B157] Otsuka T. Kawada T. Katsumata M. Ibuki C. (2006). Utility of second derivative of the finger photoplethysmogram for the estimation of the risk of coronary heart disease in the general population. *Circulation J.* 70 304–310. 10.1253/circj.70.304 16501297

[B158] Panwar M. Gautam A. Biswas D. Acharyya A. (2020). PP-Net: a deep learning framework for PPG-based blood pressure and heart rate estimation. *IEEE Sens. J.* 20 10000–10011. 10.1109/jsen.2020.2990864

[B159] Papini G. B. Fonseca P. Eerikäinen L. M. Overeem S. Bergmans J. W. Vullings R. (2018). Sinus or not: a new beat detection algorithm based on a pulse morphology quality index to extract normal sinus rhythm beats from wrist-worn photoplethysmography recordings. *Physiol. Meas.* 39:115007. 10.1088/1361-6579/aae7f8 30475748

[B160] Parak J. Korhonen I. (2014). “Evaluation of wearable consumer heart rate monitors based on photopletysmography,” in *Proceeding of the 36th Annual International Conference of the IEEE Engineering in Medicine and Biology Society*, (IEEE), 3670–3673. 10.1109/EMBC.2014.6944419 25570787

[B161] Park K. S. Choi S. H. (2019). Smart technologies toward sleep monitoring at home. *Biomed. Eng. Lett.* 9 73–85. 10.1007/s13534-018-0091-2 30956881 PMC6431329

[B162] Patterson J. A. Yang G.-Z. (2011). Ratiometric artifact reduction in low power reflective photoplethysmography. *IEEE Trans. Biomed. Circuits Syst.* 5 330–338. 10.1109/TBCAS.2011.2161304 23851947

[B163] Pereira T. Tran N. Gadhoumi K. Pelter M. M. Do D. H. Lee R. J. (2020). Photoplethysmography based atrial fibrillation detection: a review. *NPJ Digital Med.* 3 1–12. 10.1038/s41746-019-0207-9 31934647 PMC6954115

[B164] Pimentel M. A. Charlton P. H. Clifton D. A. (2015). “Probabilistic estimation of respiratory rate from wearable sensors,” in *Wearable Electronics Sensors*, ed. Mukhopadhyay S. C. (Berlin: Springer), 241–262. 10.1007/s41669-021-00290-7

[B165] Pintavirooj C. Ni B. Chatkobkool C. Pinijkij K. (2021). Noninvasive portable hemoglobin concentration monitoring system using optical sensor for anemia disease. *Healthcare* 9:647. 10.3390/healthcare9060647 34072533 PMC8230267

[B166] Poh M.-Z. Poh Y. C. Chan P.-H. Wong C.-K. Pun L. Leung W. W.-C. (2018). Diagnostic assessment of a deep learning system for detecting atrial fibrillation in pulse waveforms. *Heart* 104 1921–1928. 10.1136/heartjnl-2018-313147 29853485

[B167] Poh M.-Z. Swenson N. C. Picard R. W. (2010). Motion-tolerant magnetic earring sensor and wireless earpiece for wearable photoplethysmography. *IEEE Trans. Inform. Technol. Biomed.* 14 786–794. 10.1109/TITB.2010.2042607 20172836

[B168] Poon C. Zhang Y. (2005). “Cuff-less and noninvasive measurements of arterial blood pressure by pulse transit time,” in *Proceeding of the IEEE Engineering in Medicine and Biology 27th Annual Conference*, (IEEE), 5877–5880. 10.1109/IEMBS.2005.1615827 17281597

[B169] Poon C. C. Y. Teng X. F. Wong Y. M. Zhang C. Zhang Y. T. (2004). “Changes in the photoplethysmogram waveform after exercise,” in *Proceeding of the 2nd IEEE/EMBS International Summer School on Medical Devices and Biosensors*, (IEEE), 115–118.

[B170] Pradhan N. Rajan S. Adler A. (2019). Evaluation of the signal quality of wrist-based photoplethysmography. *Physiol. Meas.* 40:065008. 10.1088/1361-6579/ab225a 31100748

[B171] Proesmans T. Mortelmans C. Van Haelst R. Verbrugge F. Vandervoort P. Vaes B. (2019). Mobile phone–based use of the photoplethysmography technique to detect atrial fibrillation in primary care: diagnostic accuracy study of the FibriCheck app. *JMIR mHealth uHealth* 7:e12284. 10.2196/12284 30916656 PMC6456825

[B172] Rakshit R. Reddy V. R. Deshpande P. (2016). “Emotion detection and recognition using HRV features derived from photoplethysmogram signals,” in *Proceedings of the 2nd workshop on Emotion Representations and Modelling for Companion Systems* (New York, NY: Association for Computing Machinery), 1–6.

[B173] Ravichandran V. Murugesan B. Balakarthikeyan V. Ram K. Preejith S. Joseph J. (2019). “RespNet: a deep learning model for extraction of respiration from photoplethysmogram,” in *Proceeding of the 41st Annual International Conference of the IEEE Engineering in Medicine and Biology Society (EMBC)*, (IEEE), 5556–5559. 10.1109/EMBC.2019.8856301 31947114

[B174] Raza A. Tran K. P. Koehl L. Li S. (2021). Designing ECG monitoring healthcare system with federated transfer learning and explainable AI. *arXiv Preprint* ISBN:2105.12497

[B175] Reddy K. A. George B. Kumar V. J. (2008). Use of fourier series analysis for motion artifact reduction and data compression of photoplethysmographic signals. *IEEE Trans. Instrument. Meas.* 58 1706–1711.

[B176] Reisner A. Shaltis P. A. Mccombie D. Asada H. H. (2008). Utility of the photoplethysmogram in circulatory monitoring. *Anesthesiol.: J. Am. Soc. Anesthesiol.* 108 950–958. 10.1097/ALN.0b013e31816c89e1 18431132

[B177] Reiss A. Indlekofer I. Schmidt P. Van Laerhoven K. (2019). Deep PPG: large-scale heart rate estimation with convolutional neural networks. *Sensors* 19:3079. 10.3390/s19143079 31336894 PMC6679242

[B178] Reynolds K. De Kock J. Tarassenko L. Moyle J. (1991). Temperature dependence of LED and its theoretical effect on pulse oximetry. *Br. J. Anaesthesia* 67 638–643. 10.1093/bja/67.5.638 1751282

[B179] Rojano J. F. Isaza C. V. (2016). Singular value decomposition of the time-frequency distribution of PPG signals for motion artifact reduction. *Int. J. Signal Process. Syst.* 4 475–482. 10.18178/ijsps.4.6.475-482

[B180] Roy M. S. Bag P. Gupta R. (2019). “Reconstruction of corrupted and lost segments from photoplethysmographic data using recurrent neural network,” in *Proceeding of the IEEE Region 10 Symposium (TENSYMP)*, (IEEE), 214–219.

[B181] Rubins U. Grabovskis A. Grube J. Kukulis I. (2008). “Photoplethysmography Analysis of Artery Properties in Patients with Cardiovascular Diseases,” in *Proceeding of the 14th Nordic-Baltic Conference on Biomedical Engineering and Medical Physics*, (Berlin: Springer), 319–322. 10.1007/978-3-540-69367-3_85

[B182] Ruggiero E. Alonso-De Castro S. Habtemariam A. Salassa L. (2016). Upconverting nanoparticles for the near infrared photoactivation of transition metal complexes: new opportunities and challenges in medicinal inorganic photochemistry. *Dalton Trans.* 45 13012–13020. 10.1039/c6dt01428c 27482656

[B183] Rybynok V. Kyriacou P. (2010). “Beer-lambert law along non-linear mean light pathways for the rational analysis of photoplethysmography,” in *Proceeding of the Journal of Physics: Conference Series*, (IOP Publishing), 012061. 10.1088/1742-6596/238/1/012061

[B184] Saganowski S. Kazienko P. Dzieżyc M. Jakimów P. Komoszyńska J. Michalska W. (2020). Review of consumer wearables in emotion, stress, meditation, sleep, and activity detection and analysis. *arXiv preprint* ISBN:2005.00093

[B185] Salehizadeh S. Dao D. K. Chong J. W. Mcmanus D. Darling C. Mendelson Y. (2014). Photoplethysmograph signal reconstruction based on a novel motion artifact detection-reduction approach. Part II: motion and noise artifact removal. *Ann. Biomed. Eng.* 42 2251–2263. 10.1007/s10439-014-1030-8 24823655

[B186] Sanjana K. Sowmya V. Gopalakrishnan E. Soman K. (2020). Explainable artificial intelligence for heart rate variability in ECG signal. *Healthcare Technol. Lett.* 7:146. 10.1049/htl.2020.0033 33425369 PMC7787999

[B187] Sañudo B. De Hoyo M. Muñoz-López A. Perry J. Abt G. (2019). Pilot study assessing the influence of skin type on the heart rate measurements obtained by photoplethysmography with the apple watch. *J. Med. Syst.* 43 1–8. 10.1007/s10916-019-1325-2 31119387

[B188] Schäfer A. Vagedes J. (2013). How accurate is pulse rate variability as an estimate of heart rate variability?: a review on studies comparing photoplethysmographic technology with an electrocardiogram. *Int. J. Cardiol.* 166 15–29. 10.1016/j.ijcard.2012.03.119 22809539

[B189] Scholkmann F. Boss J. Wolf M. (2012). An efficient algorithm for automatic peak detection in noisy periodic and quasi-periodic signals. *Algorithms* 5 588–603.

[B190] Schrumpf F. Frenzel P. Aust C. Osterhoff G. Fuchs M. (2021a). “Assessment of deep learning based blood pressure prediction from PPG and rPPG signals,” in *Proceedings of the IEEE/CVF Conference on Computer Vision and Pattern Recognition*, (IEEE), 3820–3830.

[B191] Schrumpf F. Frenzel P. Aust C. Osterhoff G. Fuchs M. (2021b). Assessment of non-invasive blood pressure prediction from PPG and rPPG Signals Using Deep Learning. *Sensors* 21:6022. 10.3390/s21186022 34577227 PMC8472879

[B192] Seitsonen E. R. J. Korhonen I. K. J. Van Gils M. J. Huiku M. Lötjönen J. M. P. Korttila K. T. (2005). EEG spectral entropy, heart rate, photoplethysmography and motor responses to skin incision during sevoflurane anaesthesia. *Acta Anaesthesiol. Scandinavica* 49 284–292. 10.1111/j.1399-6576.2005.00654.x 15752389

[B193] Selvaraj N. Mendelson Y. Shelley K. H. Silverman D. G. Chon K. H. (2011). “Statistical approach for the detection of motion/noise artifacts in Photoplethysmogram,” in *Proceeding of the Annual International Conference of the IEEE Engineering in Medicine and Biology Society*, (IEEE), 4972–4975. 10.1109/IEMBS.2011.6091232 22255454

[B194] Senay L. C. Jr. Prokop L. D. Cronau L. Hertzman A. B. (1963). Relation of local skin temperature and local sweating to cutaneous blood flow. *J. Appl. Physiol.* 18 781–785. 10.1152/jappl.1963.18.4.781 13987937

[B195] Seok H. S. Choi B.-M. Noh G.-J. Shin H. (2019). Postoperative pain assessment model based on pulse contour characteristics analysis. *IEEE J. Biomed. Health Inform.* 23 2317–2324. 10.1109/JBHI.2018.2890482 30605112

[B196] Seyedtabaii S. Seyedtabaii L. (2008). Kalman filter based adaptive reduction of motion artifact from photoplethysmographic signal. *World Acad. Sci. Eng. Technol..* 37 173–176. 20980715

[B197] Shafique M. Kyriacou P. A. Pal S. (2012). Investigation of photoplethysmographic signals and blood oxygen saturation values on healthy volunteers during cuff-induced hypoperfusion using a multimode PPG/SpO 2 sensor. *Med. Biol. Eng. Comput.* 50 575–583. 10.1007/s11517-012-0910-z 22555629

[B198] Shan S.-M. Tang S.-C. Huang P.-W. Lin Y.-M. Huang W.-H. Lai D.-M. (2016). “Reliable PPG-based algorithm in atrial fibrillation detection,” in *Proceeding of the IEEE Biomedical Circuits and Systems Conference (BioCAS)*, (IEEE), 340–343.

[B199] Shelley K. H. (2007). Photoplethysmography: beyond the calculation of arterial oxygen saturation and heart rate. *Anesthesia Analgesia* 105 S31–S36. 10.1213/01.ane.0000269512.82836.c9 18048895

[B200] Shelley K. H. Jablonka D. H. Awad A. A. Stout R. G. Rezkanna H. Silverman D. G. (2006). What is the best site for measuring the effect of ventilation on the pulse oximeter waveform? *Anesthesia Analgesia* 103 372–377. 10.1213/01.ane.0000222477.67637.17 16861419

[B201] Shelley K. H. Murray W. B. Chang D. (1997). Arterial–pulse oximetry loops: a new method of monitoring vascular tone. *J. Clin. Monit.* 13 223–228. 10.1023/a:1007361020825 9269615

[B202] Shelley K. H. Silverman D. G. Shelley A. J. (2014). *Volume Status Monitor: Peripheral Venous Pressure, Hypervolemia and Coherence Analysis. U.S. Patent No. 8,727,997*. Washington, DC: U.S. Patent and Trademark Office.

[B203] Shi P. Hu S. Zhu Y. Zheng J. Qiu Y. Cheang P. (2009). Insight into the dicrotic notch in photoplethysmographic pulses from the finger tip of young adults. *J. Med. Eng. Technol.* 33 628–633. 10.3109/03091900903150980 19848856

[B204] Shin H. Min S. D. (2017). Feasibility study for the non-invasive blood pressure estimation based on ppg morphology: normotensive subject study. *Biomed. Eng. Online* 16:10. 10.1186/s12938-016-0302-y 28086939 PMC5234121

[B205] Shin H. S. Lee C. Lee M. (2009). Adaptive threshold method for the peak detection of photoplethysmographic waveform. *Comput. Biol. Med.* 39 1145–1152. 10.1016/j.compbiomed.2009.10.006 19883905

[B206] Shin H. S. Lee C. Lee M. (2010). Ideal filtering approach on DCT domain for biomedical signals: index blocked DCT filtering method (IB-DCTFM). *J. Med. Syst.* 34 741–753. 10.1007/s10916-009-9289-2 20703930

[B207] Sim J. K. Ahn B. Doh I. (2018). A contact-force regulated photoplethysmography (PPG) platform. *AIP Adv.* 8:045210. 10.1063/1.5020914

[B208] Simek J. Wichterle D. Melenovsky V. Malik J. Svacina S. Widimsky J. (2005). Second derivative of the finger arterial pressure waveform: an insight into dynamics of the peripheral arterial pressure pulse. *Physiol. Res.* 54:505. 15641931

[B209] Sinex J. E. (1999). Pulse oximetry: principles and limitations. *Am. J. Emergency Med.* 17 59–66. 10.1016/s0735-6757(99)90019-0 9928703

[B210] Song J. Li D. Ma X. Teng G. Wei J. (2019). PQR signal quality indexes: a method for real-time photoplethysmogram signal quality estimation based on noise interferences. *Biomed. Signal Process. Control* 47 88–95. 10.1016/j.bspc.2018.05.020

[B211] Spigulis J. Gailite L. Lihachev A. (2007a). “Multi-wavelength photoplethysmography for simultaneous recording of skin blood pulsations at different vascular depths,” in *Proceedings of the Advanced Biomedical and Clinical Diagnostic Systems V*, eds Raghavachari R. Vo-Dinh T. Grundfest W. S. Benaron D. A. Cohn G. E. (Bellingham, WA: International Society for Optics and Photonics), 64301.

[B212] Spigulis J. Gailite L. Lihachev A. Erts R. (2007b). Simultaneous recording of skin blood pulsations at different vascular depths by multiwavelength photoplethysmography. *Appl. Optics* 46 1754–1759. 10.1364/ao.46.001754 17356618

[B213] Stern R. M. (1974). Ear lobe photoplethysmography. *Psychophysiology* 11 73–75. 4810439 10.1111/j.1469-8986.1974.tb00824.x

[B214] Struys M. Vanpeteghem C. Huiku M. Uutela K. Blyaert N. Mortier E. (2007). Changes in a surgical stress index in response to standardized pain stimuli during propofol–remifentanil infusion. *Br. J. Anaesthesia* 99 359–367. 10.1093/bja/aem173 17609248

[B215] Sukor J. A. Redmond S. Lovell N. (2011). Signal quality measures for pulse oximetry through waveform morphology analysis. *Physiol. Meas.* 32:369. 10.1088/0967-3334/32/3/008 21330696

[B216] Takazawa K. (1993). Clinical usefulness of the second derivative of a plethysmogram (acceleration plethysmogram). *J. Cardiol.* 23 207–217.

[B217] Takazawa K. Tanaka N. Fujita M. Matsuoka O. Saiki T. Aikawa M. (1998). Assessment of vasoactive agents and vascular aging by the second derivative of photoplethysmogram waveform. *Hypertension* 32 365–370. 10.1161/01.hyp.32.2.365 9719069

[B218] Tang S.-C. Huang P.-W. Hung C.-S. Shan S.-M. Lin Y.-H. Shieh J.-S. (2017). Identification of atrial fibrillation by quantitative analyses of fingertip photoplethysmogram. *Sci. Rep.* 7:45644. 10.1038/srep45644 28367965 PMC5377330

[B219] Tang S. D. Goh Y. S. Wong M. D. Lew Y. E. (2016). “PPG signal reconstruction using a combination of discrete wavelet transform and empirical mode decomposition,” in *Proceeding of the 6th International Conference on Intelligent and Advanced Systems (ICIAS)*, (IEEE), 1–4.

[B220] Taniguchi H. Takata T. Takechi M. Furukawa A. Iwasawa J. Kawamura A. (2021). Explainable artificial intelligence model for diagnosis of atrial fibrillation using holter electrocardiogram waveforms. *Int. Heart J.* 62 534–539. 10.1536/ihj.21-094 34053998

[B221] Tarvirdizadeh B. Golgouneh A. Tajdari F. Khodabakhshi E. (2018). A novel online method for identifying motion artifact and photoplethysmography signal reconstruction using artificial neural networks and adaptive neuro-fuzzy inference system. *Neural Comput. Appl*. 32 3549–3566. 10.1007/s00521-018-3767-8

[B222] Tazarv A. Levorato M. (2021). A deep learning approach to predict blood pressure from ppg signals. *arXiv Preprint* 10.1109/EMBC46164.2021.9629687 ISBN:2108.00099 34892406

[B223] Temko A. (2017). Accurate heart rate monitoring during physical exercises using PPG. *IEEE Trans. Biomed. Eng.* 64 2016–2024. 10.1109/TBME.2017.2676243 28278454

[B224] Teng X. Zhang Y.-T. (2006). The effect of applied sensor contact force on pulse transit time. *Physiol. Meas.* 27:675. 10.1088/0967-3334/27/8/002 16772666

[B225] Timimi A. A. Ali M. M. Chellappan K. (2017). A novel AMARS technique for baseline wander removal applied to photoplethysmogram. *IEEE Trans. Biomed. Circuits Syst.* 11 627–639. 10.1109/TBCAS.2017.2649940 28489546

[B226] Uçar M. K. Bozkurt M. R. Polat K. Bilgin C. (2015). “Investigation of effects of time domain features of the photoplethysmography (PPG) signal on sleep respiratory arrests,” in *Proceeding of the 23nd Signal Processing and Communications Applications Conference (SIU)*, (IEEE), 124–127.

[B227] Ushiroyama T. (2005). Assessment of chilly sensation in Japanese women with lasor Doppler fluxmetry and acceleration plethysmogram with respect to peripheral circulation. *Bull. Osaka Med. Coll.* 51 76–84.

[B228] Vadrevu S. Manikandan M. S. (2018). A robust pulse onset and peak detection method for automated PPG signal analysis system. *IEEE Trans. Instrument. Meas.* 68 807–817.

[B229] Valencell T. (2015). *Optical Heart Rate Monitoring: What You Need to Know.* Raleigh: Valencell. Diakses Dari.

[B230] Venema B. Blanik N. Blazek V. Gehring H. Opp A. Leonhardt S. (2012). Advances in reflective oxygen saturation monitoring with a novel in-ear sensor system: results of a human hypoxia study. *IEEE Trans. Biomed. Eng.* 59 2003–2010. 10.1109/TBME.2012.2196276 22547451

[B231] Wang C. Huang C. Ye S. (2014). “Noninvasive cardiac output monitoring system based on photoplethysmography,” in *Proceedings of the 2014 IEEE International Conference on Progress in Informatics and Computing* (Piscataway, NJ: IEEE), 669–673.

[B232] Wang L. Poon C. Zhang Y. (2010). The non-invasive and continuous estimation of cardiac output using a photoplethysmogram and electrocardiogram during incremental exercise. *Physiol. Meas.* 31 715–726. 10.1088/0967-3334/31/5/008 20395650

[B233] Wang G. Atef M. Lian Y. (2018). Towards a continuous non-invasive cuffless blood pressure monitoring system using PPG: systems and circuits review. *IEEE Circuits Syst. Magazine* 18 6–26. 10.1109/mcas.2018.2849261

[B234] Wang K. Xu L. Wang L. Li Z. Li Y. (2003). “Pulse baseline wander removal using wavelet approximation,” in *Proceeding of the Computers in Cardiology*, (IEEE), 605–608.

[B235] Wang L. Pickwell-Macpherson E. Liang Y. P. Zhang Y. T. (2009). “Noninvasive cardiac output estimation using a novel photoplethysmogram index,” in *Proceeding of the Annual International Conference of the IEEE Engineering in Medicine and Biology Society*, (Piscataway, NJ: IEEE), 1746–1749. 10.1109/IEMBS.2009.5333091 19963762

[B236] Wannenburg J. Malekian R. (2015). Body sensor network for mobile health monitoring, a diagnosis and anticipating system. *IEEE Sens. J.* 15 6839–6852.

[B237] Webster J. G. (1997). *Design of Pulse Oximeters.* Boca Raton, FL: CRC Press.

[B238] Widrow B. Glover J. R. Mccool J. M. Kaunitz J. Williams C. S. Hearn R. H. (1975). Adaptive noise cancelling: principles and applications. *Proc. IEEE* 63 1692–1716. 10.1109/proc.1975.10036

[B239] Wong M. Y.-M. Poon C. C.-Y. Zhang Y.-T. (2009). An evaluation of the cuffless blood pressure estimation based on pulse transit time technique: a half year study on normotensive subjects. *Cardiovasc. Eng.* 9 32–38. 10.1007/s10558-009-9070-7 19381806

[B240] Xu L. Cheng J. Chen X. (2017). Illumination variation interference suppression in remote PPG using PLS and MEMD. *Electron. Lett.* 53 216–218.

[B241] Xu L. Meng M. Q.-H. Liu R. Wang K. (2008). “Robust peak detection of pulse waveform using height ratio,” in *Proceeding of the 30th Annual International Conference of the IEEE Engineering in Medicine and Biology Society*, (IEEE), 3856–3859. 10.1109/IEMBS.2008.4650051 19163554

[B242] Yan L. Hu S. Alzahrani A. Alharbi S. Blanos P. (2017). A multi-wavelength opto-electronic patch sensor to effectively detect physiological changes against human skin types. *Biosensors* 7:22. 10.3390/bios7020022 28635643 PMC5487964

[B243] Yang C. Veiga C. Rodriguez-Andina J. J. Farina J. Iniguez A. Yin S. (2019). Using PPG signals and wearable devices for atrial fibrillation screening. *IEEE Trans. Industrial Electron.* 66 8832–8842.

[B244] Yang Y. L. Seok H. S. Noh G.-J. Choi B.-M. Shin H. (2018). Postoperative pain assessment indices based on photoplethysmography waveform analysis. *Front. Physiol.* 9:1199. 10.3389/fphys.2018.01199 30210363 PMC6121033

[B245] Yousef Q. Reaz M. Ali M. A. M. (2012). The analysis of PPG morphology: investigating the effects of aging on arterial compliance. *Meas. Sci. Rev.* 12 266–271.

[B246] Yuan H. Memon S. F. Newe T. Lewis E. Leen G. (2018). Motion artefact minimization from photoplethysmography based non-invasive hemoglobin sensor based on an envelope filtering algorithm. *Measurement* 115 288–298. 10.1016/j.measurement.2017.10.060

[B247] Zangróniz R. Martínez-Rodrigo A. López M. T. Pastor J. M. Fernández-Caballero A. (2018). Estimation of mental distress from photoplethysmography. *Appl. Sci.* 8:69. 10.3390/app8010069

[B248] Zhang Q. Lindberg L.-G. Kadefors R. Styf J. (2001). A non-invasive measure of changes in blood flow in the human anterior tibial muscle. *Eur. J. Appl. Physiol.* 84 448–452. 10.1007/s004210100413 11417434

[B249] Zhang X.-Y. Zhang Y.-T. (2006). The effect of local mild cold exposure on pulse transit time. *Physiol. Meas.* 27:649. 10.1088/0967-3334/27/7/008 16705262

[B250] Zhu Q. Tian X. Wong C.-W. Wu M. (2021). Learning your heart actions from pulse: ECG waveform reconstruction from PPG. *IEEE Internet Things J.* 8 16734–16748.

[B251] Zijlstra W. Buursma A. Meeuwsen-Van Der Roest W. (1991). Absorption spectra of human fetal and adult oxyhemoglobin, de-oxyhemoglobin, carboxyhemoglobin, and methemoglobin. *Clin. Chem.* 37 1633–1638. 10.1093/clinchem/37.9.1633 1716537

[B252] Zimmermann M. Feibicke T. Keyl C. Prasser C. Moritz S. Graf B. M. (2010). Accuracy of stroke volume variation compared with pleth variability index to predict fluid responsiveness in mechanically ventilated patients undergoing major surgery. *Eur. J. Anaesthesiol. (EJA)* 27 555–561. 10.1097/EJA.0b013e328335fbd1 20035228

